# Identifying and quantifying main components of physiological noise in functional near infrared spectroscopy on the prefrontal cortex

**DOI:** 10.3389/fnhum.2013.00864

**Published:** 2013-12-17

**Authors:** Evgeniya Kirilina, Na Yu, Alexander Jelzow, Heidrun Wabnitz, Arthur M. Jacobs, Ilias Tachtsidis

**Affiliations:** ^1^Department of Education and Psychology, Dahlem Institute for Neuroimaging of Emotion, Free University of BerlinBerlin, Germany; ^2^Department of Medical Physics and Bioengineering, University College LondonLondon, UK; ^3^Physikalisch-Technische Bundesanstalt (PTB)Berlin, Germany

**Keywords:** fNIRS, physiological noise, wavelet coherence, de-noising methods

## Abstract

Functional Near-Infrared Spectroscopy (fNIRS) is a promising method to study functional organization of the prefrontal cortex. However, in order to realize the high potential of fNIRS, effective discrimination between physiological noise originating from forehead skin haemodynamic and cerebral signals is required. Main sources of physiological noise are global and local blood flow regulation processes on multiple time scales. The goal of the present study was to identify the main physiological noise contributions in fNIRS forehead signals and to develop a method for physiological de-noising of fNIRS data. To achieve this goal we combined concurrent time-domain fNIRS and peripheral physiology recordings with wavelet coherence analysis (WCA). Depth selectivity was achieved by analyzing moments of photon time-of-flight distributions provided by time-domain fNIRS. Simultaneously, mean arterial blood pressure (MAP), heart rate (HR), and skin blood flow (SBF) on the forehead were recorded. WCA was employed to quantify the impact of physiological processes on fNIRS signals separately for different time scales. We identified three main processes contributing to physiological noise in fNIRS signals on the forehead. The first process with the period of about 3 s is induced by respiration. The second process is highly correlated with time lagged MAP and HR fluctuations with a period of about 10 s often referred as *Mayer waves*. The third process is local regulation of the facial SBF time locked to the task-evoked fNIRS signals. All processes affect oxygenated haemoglobin concentration more strongly than that of deoxygenated haemoglobin. Based on these results we developed a set of physiological regressors, which were used for physiological de-noising of fNIRS signals. Our results demonstrate that proposed de-noising method can significantly improve the sensitivity of fNIRS to cerebral signals.

## Introduction

Functional Near-Infrared Spectroscopy (fNIRS) is a powerful tool to study functional organization of the prefrontal cortex (Scholkmann et al., 2013c). Due to absence of hair on the forehead, and relatively short distance between the forehead surface and the frontal cortex (Okamoto et al., 2004) this cortical area is well accessible by near-infrared light. However, despite these beneficial biophysical circumstances physiological noise generally limits overall fNIRS sensitivity and specificity on the prefrontal cortex (Tachtsidis et al., 2009; Aletti et al., 2012; Gagnon et al., 2012; Kirilina et al., 2012). Physiological fNIRS noise is induced by fluctuations in both blood volume and blood oxygenation in the extra- and intracranial tissues. Global and local blood flow regulation processes in these anatomical compartments lead to oscillations on multiple time scales. As a result fNIRS sensitivity to functional neuronal signals on the single subject level deteriorates and additional variance is added on the group level, due to inter-subject variability of the systemic and skin physiology.

In literature a number of methods were proposed for the separation of physiological noise from the cerebral activation (Saager and Berger, 2005; Katura et al., 2008; Gregg et al., 2010; Saager et al., 2011; Aletti et al., 2012). Comprehensive review of these methods can be found in Scholkmann et al. (2013c). Here we briefly summarize some of them. The majority of these approaches may be subdivided into three classes. Among them the most powerful methods are based on the idea of superficial signal regression. These methods require additional fNIRS channels with short spatial source-detector separation (Saager and Berger, 2005; Gregg et al., 2010; Saager et al., 2011; Gagnon et al., 2012; Funane et al., 2013). Nevertheless, these approaches still fail in removing physiological noise components originating from the cerebral compartment. Secondly, transient differences of functional signals and physiological noise have been exploited by a variety of methods (Kohno et al., 2007; Zhang et al., 2009, 2012b; Tanaka et al., 2013). These methods typically assume statistical independence between physiological noise and cerebral signal and fail to separate task-evoked responses of physiological parameters. In the third class, separation of forehead noise from cerebral fNIRS signals is achieved by exploring additional information from concurrent recordings of global systemic physiology, e.g., blood pressure and heart rate (HR) or local physiological parameters, e.g., skin blood flow (SBF) (Franceschini et al., 2006; Rowley et al., 2007; Tachtsidis et al., 2010; Minati et al., 2011; Patel et al., 2011; Takahashi et al., 2011; Sato et al., 2013). However, this separation is challenging, since the precise physiological fNIRS noise mechanisms are unknown. This shortcoming results from the lack of robust physiological models capable of relating physiological parameters like e.g., blood pressure or SBF with the fNIRS observable, tissue haemoglobin concentration. Further complications arise from the complex structure of physiological noise. Physiological noise involves components originating from both cerebral and extra-cerebral tissue, and global systemic as well as local tissue specific regulatory processes. Moreover different physiological processes dominate at different time scales.

The goal of the present study is to improve this situation by identifying the global systemic and local tissue-specific physiological noise processes in fNIRS recordings at the forehead and to quantify their relative impact in a group analysis.

Below we shortly review the literature on physiological processes which could potentially contribute to the physiological noise in fNIRS at different time scales. The most prominent, however, unproblematic, component is the heart pulsation with frequencies around 1 Hz, which may be effectively removed by low-pass filtering, since the corresponding frequency band is well distinguished from that of hemodynamic responses. Simple de-trending or high pass filtering also removes very slow drifts on the time scale of several minutes. The major concerns for fNIRS data quality are oscillations in the range from 0.2 to 0.005 Hz, since they account for the main components of physiological noise. Moreover, these fluctuations may in the worst case be synchronized with the task and might thereby lead to false positives in fNIRS activation maps (Tachtsidis et al., 2009).

Based on physiological considerations it is common to distinguish three distinct bands: high frequency oscillations (HFOs) ranging from 0.2 to 0.5 Hz, low frequency oscillations (LFOs) from 0.08 to 0.15 Hz and very low frequency fluctuations (VLFOs) from 0.02 to 0.08 Hz. In the following we will first focus on global systemic and then on local regulatory processes specific to cerebral or to extra-cerebral compartments.

HFOs in fNIRS signals, with frequencies around 0.3 Hz, are predominantly induced by direct or mediated influence of respiration. In the following we therefore refer to the frequency band around 0.3 Hz as R-band.

High variability in global systemic parameters of blood pressure and HR was found in the frequency band of LFOs (Julien, 2006). First reported by Mayer in 1876 (Mayer, 1876), these slow blood pressure waves are usually referred to as Mayer waves and the corresponding frequency band as M-band. In how far Mayer waves are propagated into cerebral and skin compartments remains controversial (Tong and Frederick, 2010; Tong et al., 2011).

In the cerebral compartment LFOs, independent from blood pressure fluctuations, were observed with Laser Doppler flowmetry (LDF) (Hudetz et al., 1992) and optical imaging in animals (Mayhew et al., 1996) and humans (Rayshubskiy et al., 2013) and with fNIRS on the human cortex (Elwell et al., 1999; Obrig et al., 2000; Tachtsidis et al., 2004).

Also SBF exhibits LFOs and VLFOs. LFOs in the M-band were observed even in isolated vessels (Johansson and Bohr, 1966) and were shown to be related to the activity of vessel walls. The SBF fluctuations in VLFO band originate most likely from sympathetic control of the peripheral vasculature (Kastrup et al., 1989; Söderström et al., 2003). The forehead skin vasculature constitutes a separate layer of sympathetic and parasympathetic innervations (Drummond, 1996, 1997). This fact allows for a synchronization between skin VLOFs and certain type of tasks in the forehead (Tachtsidis et al., 2009; Kirilina et al., 2012). Since the typical period of stimulation used in block design fNIRS studies often corresponds to VLFO frequency band, we refer to this band as A-band (activation) throughout this manuscript.

In summary, physiological fNIRS noise originates from different global systemic and local regulatory processes, each dominating at a different time scale. The goal of the present study was to identify the dominating processes and quantify their relative impact. We furthermore aim to develop a method to account for the contribution of each noise process in the fNIRS analysis, and therefore provide a method for physiological de-noising of fNIRS data. In order to achieve this challenging task we combined depth selective time-domain fNIRS measurements with concurrent recordings of global systemic peripheral physiological measurements of respiration, mean arterial blood pressure (MAP) and HR as well as a local SBF recordings on the forehead. Temporal disentanglement of the different physiological processes was realized by an advanced WCA. This method, originally developed in the field of geo science and meteorology (Torrence and Webster, 1999; Grinsted et al., 2004) but also applied to fNIRS signals analysis (Rowley et al., 2007; Li et al., 2010, 2013; Zhang et al., 2012a). WCA allows to target the coherent content of two temporal signals specifically at multiple time scales. Based on results of the WCA we developed a physiological de-noising method for fNIRS signals based on General Linear Modeling (GLM) (Kiebel and Holmes, 2007) and auxiliary physiological regressors (Tachtsidis et al., 2010). This approach allowed us to develop a novel versatile but yet robust physiological fNIRS de-noising method.

In the Theory section we briefly describe the theoretical background of wavelet transform and WCA used in this study to decompose physiological noise in fNIRS into components coherent with particular physiological processes. In the Methods section we describe the setup used for time-domain fNIRS experiments on the forehead. Based on the findings summarized in the Results section we discuss the possible origin of three processes contributing to the physiological noise and analyse the performance of the developed de-noising technique.

## Theory

### Wavelet transform

Continuous wavelet transform allows for constructing time-frequency representations of a signal with optimal resolution for each frequency band. Continuous wavelet transform of a time dependent signal *S*(*t*) is defined as (Mallat, 2009):

(1) $$W_{S}\left(\alpha ,t_{0}\right)=\frac{1}{\sqrt{\alpha }}\int_{-\infty }^{\infty }S(t)\psi^{*}\left(\frac{t-t_{0}}{\alpha }\right)dt$$

Where *t* and *t*_0_ are time and time shift, respectively, α is a scale (of dimension time), and *W_S_*(α, *t*_0_) represents the wavelet transform of signal *S*(*t*). ψ(*t*) is the mother wavelet function. In the present study a Morlet wavelet (Goupillaud et al., 1984) was used as mother wavelet function. This complex function may be expressed as product of a harmonic function and a Gaussian:

(2) $$\psi (s)=\frac{1}{\sqrt{\pi f_{b}}}e^{i2\pi f_{c}s}e^{-\frac{s^{2}}{f_{b}}}$$

where *f_c_* and *f_b_* are dimensionless parameters, determining the wavelet center frequency and wavelet bandwidth, respectively, and $s=\frac{t-t_{0}}{\alpha }$ is a dimensionless variable. We used *f_c_* = 1 and *f_b_* = 3, a parameter set that provides optimal trade-off between time and frequency resolution. *W_S_*(α, *t*_0_) is a complex function of scale α and position *t*_0_. If scale α is greater than one, the mother wavelet function ψ is stretched in the time domain. If α is smaller than one (but positive) the function is compressed in time. The scaling factor $\frac{1}{\sqrt{\alpha }}$ in Equation (1) is to ensure the energy normalization for all values of scale α.

From Equation (2) one can see that the relationship between wavelet scale α and pseudo-frequency *f_a_* is given by:

(3) $$f_{a}=\frac{f_{c}}{\alpha }$$

In our case the relationship between the scale α and the pseudo-frequency simplifies to $f_{a}=\frac{1}{\alpha }$.

**Figure 1:**
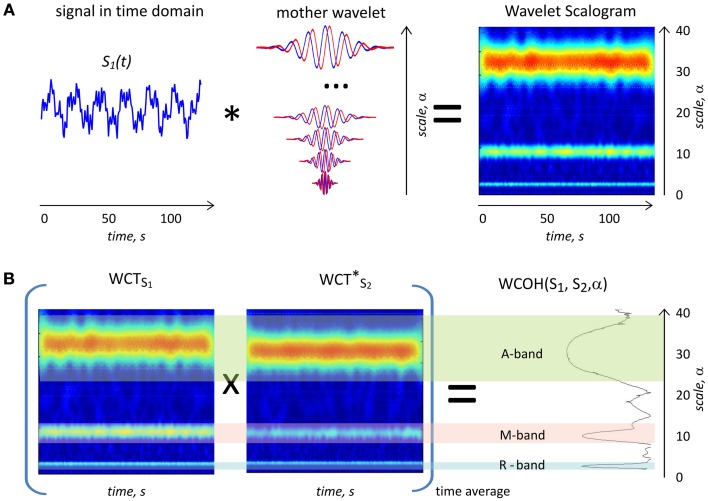
**(A)** Scheme of the wavelet transform. The synthetic signal *S*_1_(*t*) (left) is convolved with complex mother wavelet function stretched with the scale parameter α (middle) to provide 2D wavelet scalogram on the right side. The real part of mother Morlet wavelet is plotted with red and imaginary part plotted with a blue line. Sign ^*^indicates cross-correlation operation. The magnitude of the complex 2D wavelet scalogram is shown in color code. The synthetic curve *S*_2_(*t*) contains three harmonic components with the frequencies 0.3, 0.1, and 0.033 Hz, respectively, as well as additional white noise. The scalogram on the right hand side clearly demonstrates three components at scales 3, 10, and 34 s corresponding to the inverse frequencies of the signal components. **(B)** Example of a 2D wavelet coherence calculated for two synthetic signals with three coherent components. WCT_S1_ on the left and WCT^*^_S2_ in the middle are complex 2D wavelet scalograms of two signals (plotted as color coded magnitude values), both containing phase shifted coherent components with frequencies 0.3, 0.1, and 0.034 Hz, respectively, and additional non-coherent white noise. Sign × indicates scalar product operation. On the right side the magnitude value of wavelet coherence WCOH(*S*_1_, *S*_2_, α) is shown. Three peaks on the WCOH plot correspond to the three coherent components in the two signals in R-, M-, and A bands.

To exemplify the above described algorithm, the wavelet transform of a synthetic signal *S*(*t*) is outlined from left to right in Figure 1A.

The synthetic signal *S*(*t*) is a sum of three weighted sinusoidal signals with frequencies of 0.3 Hz (R-band), 0.1 Hz (M-band), and 0.034 Hz (A-band) and white Gaussian noise. The signal *S*(*t*) models a hypothetical physiological noise process with three components of different physiological origin.

The absolute values of *W_S_*(α, *t*) are depicted as 2D wavelet scalogram in the right part of Figure 1A. This representation has clear maxima at the three scales 3, 10, and 33 s, corresponding to the inverse frequencies of the components present in the signals.

Please note that the width of the large-scale (low-frequency) component is proportionally larger, than that of the low-scale (high-frequency) component. This reflects an intrinsic wavelet transform property, which adjusts the frequency resolution to the frequency band.

### Wavelet coherence analysis

WCA is a tool to evaluate the correlation of two time dependent signals separately at different time scales.

Wavelet coherence of two signals *S*_1_(*t*) and *S*_2_(*t*) is defined as follows (Torrence and Webster, 1999; Grinsted et al., 2004):

(4) $$\bar{\text{WCOH}}(\alpha )=\frac{R_{X,Y}\left(W_{S_{1}},W_{S_{2}},\alpha \right)}{\sqrt{\left|R_{X,X}\left(W_{S_{1}},\alpha \right)\cdot R_{X,X}\left(W_{S_{2}},\alpha \right)\right|}}$$

*R_X, Y_*(*W*_*S*1_, *W*_*S*2_, α) indicates the covariance or scalar product of the wavelet coefficients, *W*_*S*_1__(α, *t*_0_), and *W*_*S*_2__(α, *t*_0_) of signals *S*_1_ and *S*_2_ at scale α. *R*_*X, X*_(*W*_*S*_1__, α) and *R*_*X, X*_(*W*_*S*_2__, α) denote the autocorrelation or power of *W*_*S*_1__(α, 0) and *W*_*S*_2__(α, 0). In other words, the complex function WCOH(α) represents the cross-spectral power in two time series as a fraction of the total power of the series. The absolute value of wavelet coherence WCOH(α) ranges from 0 to 1 with 1 indicating strongest correlation. The phase of the wavelet coherence provides information about the time lag between two signals. We define a time lag between two signals at scale α as the product of α and the wavelet coherence phase at this scale (expressed in radians).

Note that instead of conventionally performed smoothing in both time and scale dimensions (Torrence and Webster, 1999), we calculated only an average over the whole measurement period, but omitted smoothing in the scale dimension.

To illustrate the ability of WCOH(α) to detect coherent content in two signals at multiple time scales, Figure 1B provides an example of WCA of two synthetic signals with coherent components in three frequency bands. Two signals *S*_1_(*t*) and *S*_2_(*t*) are hypothetical physiological signals containing coherent oscillations in R-, M-, and A-bands. From left to right 2D wavelet scalograms of *S*_1_(*t*) and *S*_2_(*t*) are plotted together with the wavelet coherence (outmost right plot). The first signal *S*_1_(*t*) is identical to the example provided in Figure 1A. The second signal *S*_2_(*t*) also contains white Gaussian noise added to the sinusoidal components. *S*_2_(*t*) contains the same frequencies as *S*_1_(*t*) (0.3, 0.1, and 0.034 Hz), but with different phases and weights for the three components.

The wavelet coherence plot in Figure 1B shows three distinct maxima at 3, 10, and 34 s (equal to inverse frequencies of signal components), corresponding to three coherent components contained in the signals *S*_1_(*t*) and *S*_2_(*t*). In contrast to a simple correlation analysis WCOH(α) provides detailed frequency information about the coherent content in both signals.

In the current study we explore this property of WCA in order to analyze coherence between global systemic and local physiological processes and fNIRS signals at different time scales. In this way we are able to decompose physiological noise in fNIRS into components induced by several physiological processes.

Note the different widths of the maxima at the three different scales in the Figure 1B. Due to the wavelet transform properties, the frequency resolution degrades with the increasing scale α. However, the ratio between the resolution and α is independent of α.

## Methods

fNIRS and concurrently recorded physiological signals were acquired as part of a comparative fNIRS/fMRI experiment previously reported in (Kirilina et al., 2012). Here we only briefly describe one part of experimental settings including fNIRS and peripheral physiological measurements, which were used in the analysis presented. A detailed description of subject population, stimulation paradigm, NIRS instrumentation and fNIRS data pre-processing can be found in (Kirilina et al., 2012).

### Subjects

Fifteen healthy subjects (5 female, age 34.9 ± 7.2 years) took part in the present study. Due to technical reasons data from one subject was excluded from further analysis. During the experiment subjects were sitting in upright position in front of a computer monitor, while responding with right hand button presses. All subjects gave their informed consent to the experimental protocol, which was approved by a local ethics committee.

### Stimulus

A variation of continuous performance task (CPT) combined with a semantic categorization task was used to achieve the cerebral activation in the frontal lobe (bilateral Brodmann Area 10). A series of German words representing either concrete or abstract categories were continuously presented to the subjects in semantic (sem-CPT) and control (word-CPT) tasks. In sem-CPT the subjects were instructed to press the left button if a concrete word was presented after an abstract word. In any other case, they should press the right button. In word-CPT the subjects were instructed to press the left button if one particular target word 1 (VORZUG, German for “preference”) followed target word 2 (KOFFER, German for “suitcase”), and right button in any other case. Both tasks were performed in nine 34.15 s long blocks in alternating order, interleaved by 31.11 s long baseline blocks. During the baseline blocks a fixation cross was presented in the middle of the screen. Pre- and post-baselines of 120 s were recorded for each subject.

### Data acquisition

**Figure 2:**
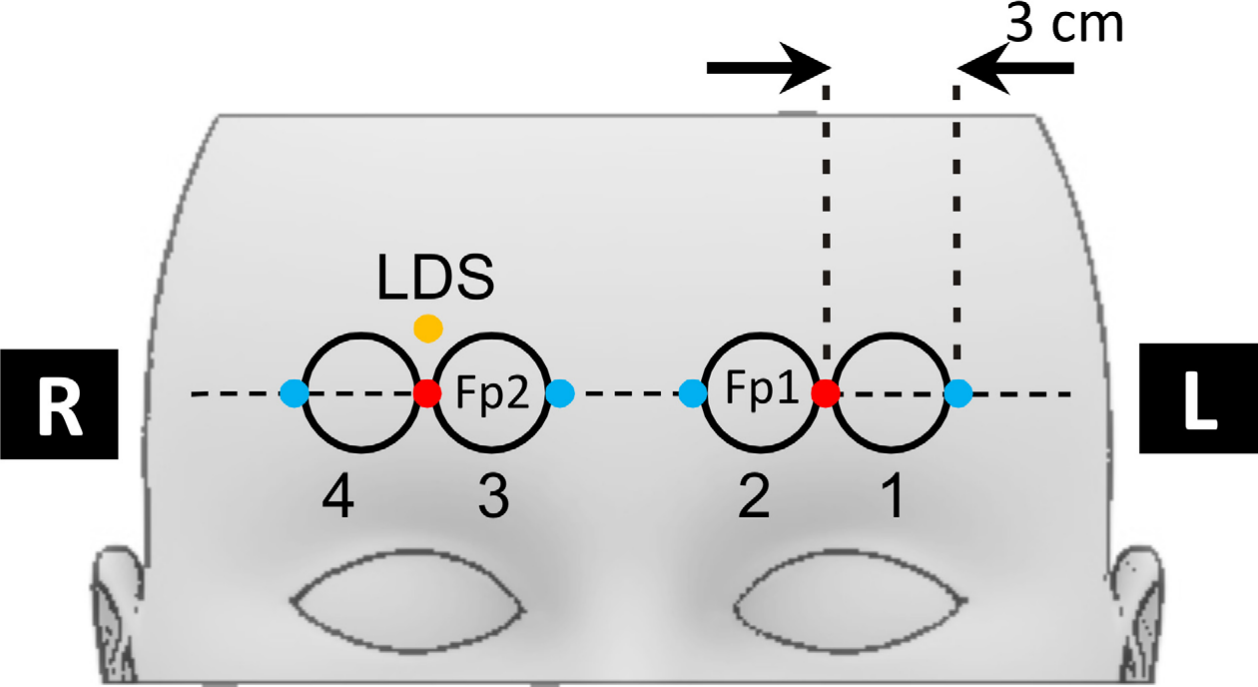
**Scheme of the fNIRS optode placement on the forehead**. Red dots indicate the positions of fNIRS source optodes, blue dots the positions of the four detector optodes. Numbered circles indicate the locations of the four fNIRS channels. The orange dot indicates the positions of the laser Doppler sensor.

Concentration changes in oxygenated and deoxygenated haemoglobin, in the following referred as ΔHbO and ΔHbR, respectively, were measured by the PTB time-domain optical brain imager (Wabnitz et al., 2005, 2010). This device provides three wavelengths 689, 797, and 828 nm. The laser power was split to obtain two sources. Diffusely reflected light was collected by four detection fiber bundles and detected by fast photomultipliers connected to a multi-board time-correlated single photon counting (TCSPC) system. Distributions of photon time of flight (DTOFs) were acquired with time bins of 24.4 ps width and at a 20 Hz rate. A set of one source fiber and two detection fiber bundles was placed on the left and right forehead, respectively, along the Fp1–Fp2 line defined by the international 10–20 system, as illustrated in Figure 2. A source-detector separation of 3 cm was chosen for all fNIRS channels. To exploit the potential of time-domain fNIRS for the separation of extracranial and cerebral signals, the measured DTOFs were analyzed in terms of statistical moments (Liebert et al., 2004). ΔHbO and ΔHbR time courses were extracted from changes in three different measures. These were (i) the 0th moment of DTOF, *m*_0_, corresponding to the total photon count, (ii) the 1st moment of DTOF, *m*_1_, i.e., the photon mean time of flight and (iii) the 2nd central moment of DTOF, the variance *V*. A detailed description of the procedure is given in Appendix 1 of (Kirilina et al., 2012). We would like to note that haemoglobin concentration changes based on *m*_0_ are analogous to signals measured in conventional continuous wave (cw) fNIRS experiments. ΔHbO and ΔHbR based on *m*_1_ and on the variance signal *V* have the advantage to be more sensitive to deeper and less sensitive to superficial absorption changes (Liebert et al., 2004; Wabnitz et al., 2005).

### Peripheral physiology

Alongside with fNIRS recordings, the following global and local physiological signals were recorded: HR (from electrocardiography ECG), blood pressure, scalp blood flow, and respiration chest movement.

Respiration (RSP) and ECG were recorded using a Nexus-10 system (Mind Media, The Netherlands). The respiration was recorded with a Hall-sensor based respiratory belt placed over the subject's lower ribs. ECG was recorded with a sampling rate of 256 Hz, using two electrodes placed on the upper right and lower left part of the chest, respectively, and a ground electrode placed on the upper left chest.

HR was defined for each heartbeat as the inverse time interval between two subsequent R-peaks in the ECG time trace. A PortaPress system (TNO TPD Biomedical Instrumentation) was used to continuously measure MAP at the left hand index finger. HR and MAP signals originally measured for time points corresponding to heartbeats were re-sampled onto the equidistant 1 Hz time grid.

Changes in SBF were recorded by a floLAB Laser Doppler Perfusion Monitor (Moor Instruments) operating at an emitter-detector distance of about 1 mm. The laser Doppler probe was placed on the right forehead 15 mm above the NIRS source (see Figure 2). Due to the close placement of fNIRS detectors and laser Doppler probe the light emitted by the floLAB device was detectable by the td fNIRS system. This light appeared as a constant uncorrelated background in the td-NIRS measurement and was subtracted during the data preprocessing procedure. The additional contribution of the background to photon noise was insignificant.

### Data analysis

#### Pre-processing

Pre-processing, wavelet analysis and GLM analysis of fNIRS and physiological data was performed by own software written in MATLAB (R2012a, Mathworks Inc.). All signals were first filtered by a low-pass filter, with a cutoff frequency of 0.8 Hz to remove the variability due to the cardiac cycle. In addition, a high-pass filter with a cutoff frequency of 0.008 Hz was used to remove very slow signal and baseline shift variations (Mitsis et al., 2004). In both cases fifth order Butterworth filters in forward and backward direction were used for further data filtering. After filtering, all recorded measurements were down sampled to 1 Hz.

#### Wavelet coherence analysis

After preprocessing of ΔHbO and ΔHbR signals from four channels and three moments as well as the time dependent physiological traces for each subject, WCA was employed to investigate the coherence between physiological noise in fNIRS and peripheral physiological traces.

**Figure 3:**
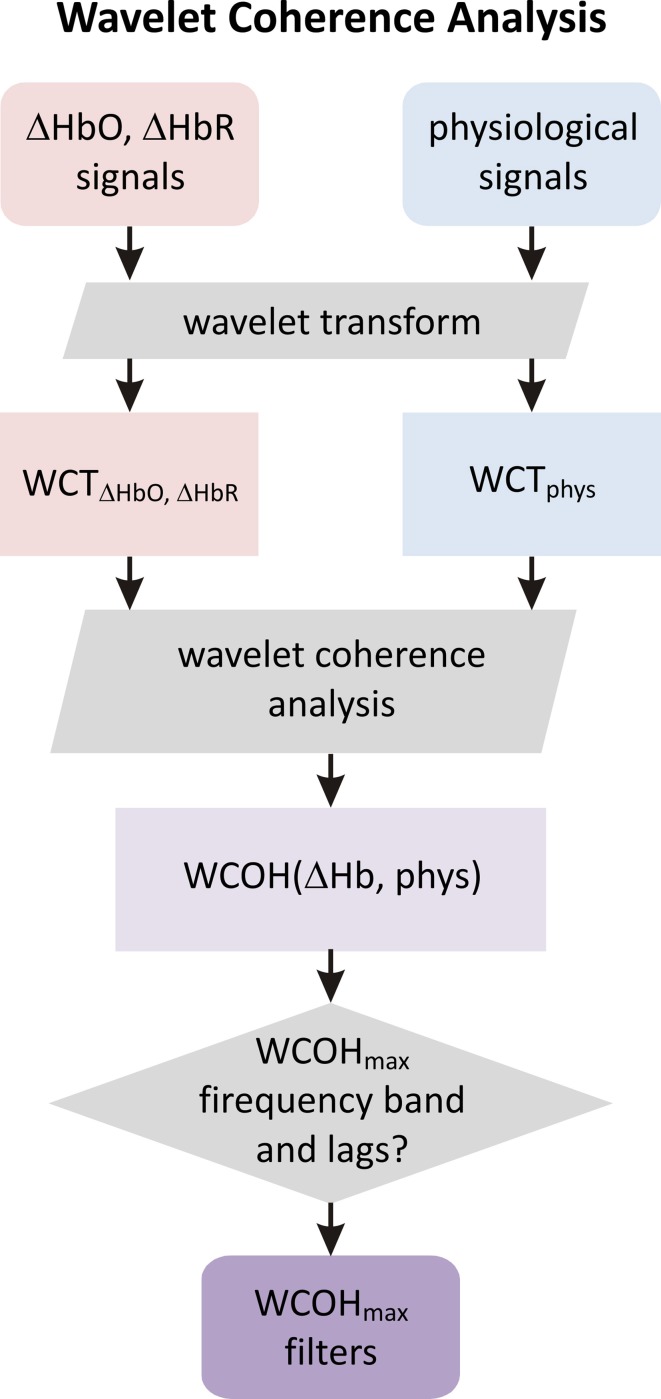
**Scheme of wavelet coherence analysis (see Section Wavelet Coherence Analysis for details)**. In the first step fNIRS and physiological signals are wavelet transformed to provide wavelet scalograms WCT_HbO, HbR_ and WCT_phys_. The coherence between these scalograms yields a scale dependent wavelet coherence. Frequency bands corresponding to the maximum of the wavelet coherence and the coherence phase at maximum are determined. These parameters are further used to create auxiliary physiological regressors in the GLM de-noising (see Section General Linear Modeling and Physiological De-noising and Figure 4).

We used a complex Morlet mother wavelet as defined by function *cmor3-1*, [see Equation (2)] in the MATLAB Wavelet Toolbox. A complex wavelet transform was performed on an equidistant scale grid ranging from 0.2 to 50 s with 0.2 s steps, corresponding to pseudo-frequencies from 5 Hz down to 0.02 Hz. For each subject, wavelet coherence was calculated between four physiological traces and twelve haemoglobin concentration changes measured for the four channels and based on three moments. Magnitude and phase of the group average was calculated in the next step for each pair of signals. Based on the observed maxima of the magnitude of the group wavelet coherence we identified three bands. Mean phase differences and corresponding time lags between each signal pair were calculated within each scale band. The above described procedure of WCA between fNIRS and physiological signals is illustrated as a flowchart in Figure 3.

Both fNIRS and physiological signals contain a certain amount of measurement noise. Therefore, the obtained wavelet coherence values include a stochastic component induced by experimental noise. The noise propagation in wavelet coherence is not linear due to the presence of the denominator in Equation (4). Therefore, proper statistical analysis of the group average wavelet coherence values and their comparison becomes a highly complex challenge. This situation is further complicated by the fact that fNIRS signals based on different moments have different photon noise contributions and that the physiological traces have different frequency spectra.

In order to overcome this non-linear problem, we numerically estimated the impact of different levels of photon noise and uncorrelated physiological noise components on the group wavelet coherence values. This was achieved by adding artificial noise to experimental data. In the following the numerical procedure to estimate the influence of these noise components is described in detail.

***Influence of photon noise on wavelet coherence***. In order to be able to correctly compare wavelet coherences obtained for fNIRS signals based on different moments, their different levels of photon noise were taken into account. In general, photon noise has increasing influence for haemoglobin concentrations based on higher order moments. We first theoretically estimated the standard deviation of the photon noise component in the measured ΔHbO and ΔHbR signals based on the three different moments. Theoretical and numerical details of this estimation are described in the Appendix. In a second step we performed numerical experiments, adding a controlled amount of synthetic white Gaussian noise to the (least noisy) measured *m*_0_-based ΔHbO and ΔHbR signals. The noise amplitudes were chosen in a way, that the photon noise in the synthetic *m*_0_-based signal matched the level of photon noise in *m*_1_- and *V*-based ΔHbO and ΔHbR signals, respectively. The group wavelet coherence, between synthetic “noise matched” signals and physiological traces, was calculated and compared to the wavelet coherence obtained for experimentally measured *m*_1_- and *V*-based signals.

***Uncorrelated physiological noise and wavelet coherence***. Furthermore, we numerically estimated the noise levels of the magnitude of group wavelet coherence obtained for uncorrelated physiological noise. For this purpose we calculated the wavelet coherence on the single subject level, between the fNIRS signals measured for each subject and physiological signals measured for different randomly chosen subjects. Additionally we circularly shifted the physiological traces with a random time shift, different for each subject in order to avoid inter-subject coherence effects potentially induced by the task. The wavelet coherences of individual subjects were subjected to a group analysis by calculating the absolute value of the group mean wavelet coherence value.

#### General linear modeling and physiological de-noising

As was described above, the proper statistical analysis of group wavelet coherence is mathematically challenging. Therefore, in this study we used the results of the group WCA only in a qualitative manner. Based on the maxima in wavelet coherence we identified the main processes contributing to physiological noise in fNIRS. We then used this information in order to create a set of auxiliary regressors for physiological noise modeling as described in the next session. Finally with the help of the GLM analysis informed by WCA we were able to statistically analyze the impact of each physiological process at the group level.

The GLM analysis was performed on the fNIRS signal time traces from the entire experimental sessions. Our model to analyze fNIRS signals included one regressor modeling task-related brain activation and auxiliary regressors modeling physiological noise. To create a regressor modeling task-related brain activation, boxcar functions of the task presentation were convolved with the standard haemodynamic response function as it is implemented in SPM8 software package (Friston and Stephan, 2007). The physiological noise regressors were constructed based on the results of the WCA.

For each physiological trace we determined a frequency band demonstrating maximal wavelet coherence with fNIRS signals. The time delay between fNIRS and physiological signals was determined in these bands, based on the phase of the group averages of wavelet coherence.

**Figure 4:**
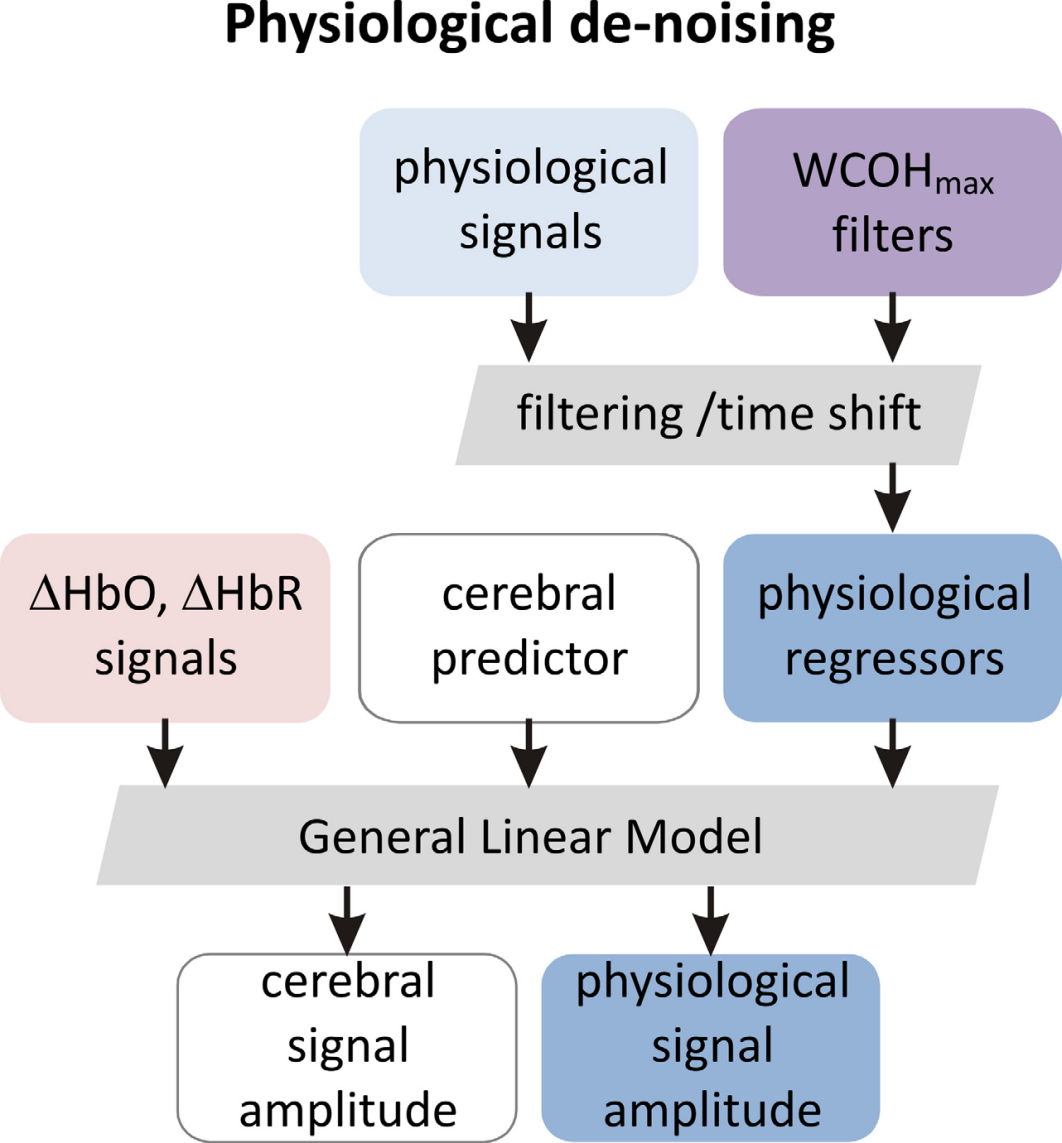
**The scheme of de-noising GLM analysis (see Section General Linear Modeling and Physiological De-noising for details)**. In the first step physiological signals are filtered and time shifted using frequency bands and time shifts determined in the group wavelet coherence analysis as described in Section Section Wavelet Coherence Analysis and illustrated in Figure 3. In this way the set of auxiliary physiological regressors is created. This set is completed with predictors modeling cerebral activation and is used in the GLM analysis (see Section General Linear Modeling and Physiological De-noising and this figure).

Physiological signals of the entire experimental session of each participant were then band-pass filtered at these frequency bands using a fifth order Butterworth filter in forward and backward direction. After filtering the signals were time shifted by the delay determined as described before. Filtered and time-shifted signals were used as regressors in the GLM analysis. The above described GLM de-noising procedure is illustrated as a flowchart in Figure 4.

Signal amplitudes corresponding to each regressor, obtained in the first level analysis for each subject, were subjected to a second level analysis, by calculating one-sample *T*-tests. For those values, which significantly differed from zero, with a significance level of *p* = 0.05 the group mean and standard deviation were calculated.

## Results

### Wavelet coherence analysis

**Figure 5:**
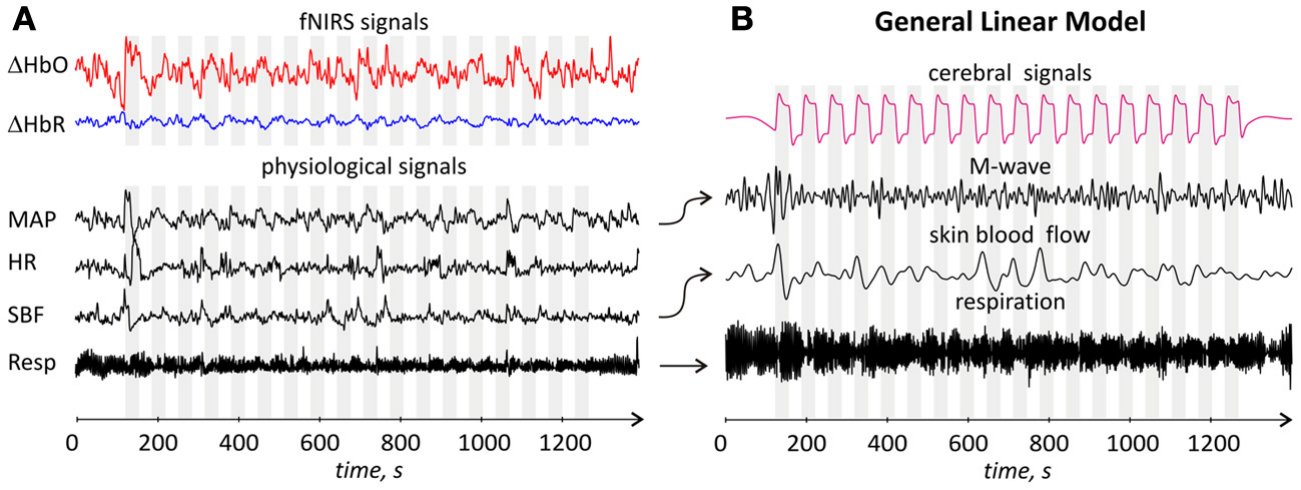
**(A)** Time traces of fNIRS and physiological signals for one representative subject. From top to bottom: single channel (channel 1) *m*_0_-based ΔHbO and ΔHbR, MAP, HR, SBF, and respiration signals. All signals are arbitrary scaled. Gray areas correspond to the durations of the stimulation blocks; **(B)** Time traces of regressors used in de-noising GLM analysis. From top to bottom: cerebral regressor, M-wave regressor obtained by filtering and time shifting of the MAP time trace, skin blood flow and respiratory regressors obtained by appropriate filtering and time shifting of skin blood flow and respiration traces, respectively. The arrows between **(A,B)** parts indicate which signals were used to generate the corresponding regressor.

Figure 5A shows time traces of the single channel ΔHbO and ΔHbR signals, as well as time traces of four physiological signals for one representative subject.

**Figure 6:**
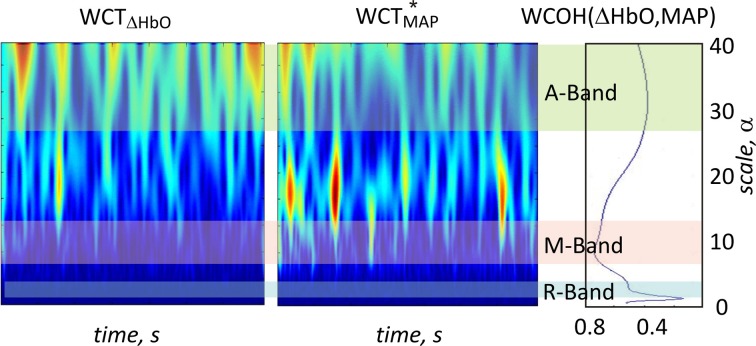
**Example of magnitude 2D wavelet scalograms (left ΔHbO signal, middle MAP signal) and magnitude of the wavelet coherence between ΔHbO and MAP (right) for one representative subject**. Sign ^*^indicates complex conjugation. Light blue, light red and light green bars indicate the following three scale ranges: R-band (around 3 s, 0.3 Hz), M-band (around 10 s, 0.1 Hz) and A-band (around 35 s, 0.029 Hz), respectively. The peaks in wavelet coherence are observed in R- and M-bands for this subject.

Figure 6 shows the 2D wavelet scalograms of ΔHbO and MAP and wavelet coherence between these two signals for one channel of a representative subject. In this particular case an increased coherence is observed for scales around 10 s, corresponding to the M-band.

**Figure 7:**
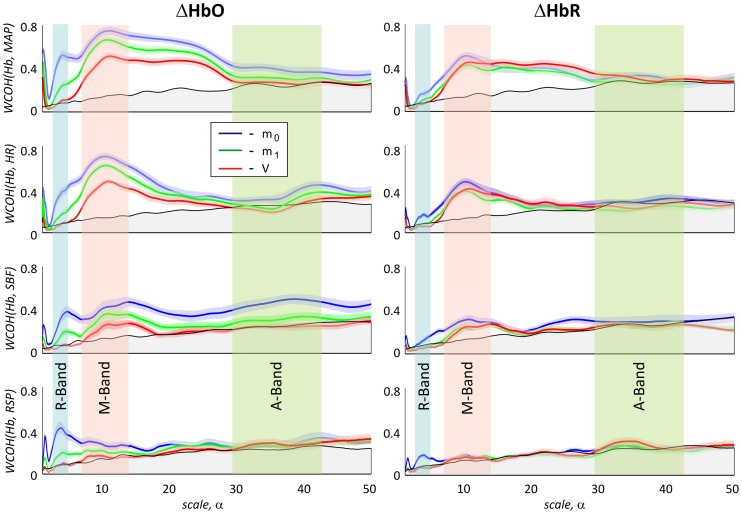
**Magnitude values of group average of wavelet coherence between haemoglobin concentration changes ΔHbO (left) and ΔHbR (right) and the four physiological signals**. In the columns from top to bottom results for mean arterial blood pressure, heart rate, skin blood flow, and respiration are shown, respectively. Haemoglobin concentration changes extracted from m_0_, m_1_, and V are plotted in blue, green, and red, respectively. The gray area under the black curve indicates the noise level for the magnitude of the group mean value of the WCOH between ΔHbO or ΔHbR and the physiological signals. Light blue, light, red and light green bars indicate the following three time scale ranges: R-band (scales around 3 s and pseudofrequencies around 0.3 Hz), M-band (scales around 10 s and pseudo-frequencies around 0.1 Hz) and A-band (scales around 35 s and pseudo-frequencies around 0.033 Hz), respectively. The colored shadowed areas represent standard error of mean for each curve.

Figure 7 depicts absolute values of the group average of the wavelet coherence between transient haemoglobin concentrations, e.g., (ΔHbO and ΔHbR) and the physiological signals MAP, HR, SBF, and RSP, respectively. The black solid curves in each subplot in Figure 7 indicate the level of coherency obtained between corresponding fNIRS signal and uncorrelated physiological noise as described in section Uncorrelated Physiological Noise and Wavelet Coherence. Therefore, all values within gray area under the black curve can be considered as not significant.

There are three distinct peaks of the wavelet coherence between fNIRS and physiological signals within three different frequency bands. The peak with the lowest scale around 3 s (corresponding to the pseudo-frequency of 0.33 Hz in R-band) is most clearly observed for the respiration signal, but also present in the wavelet coherence with MAP, HR, and SBF.

The peak with scale in the range of about 10 s (corresponding to the pseudo-frequency of 0.1 Hz in M-band) is strongly present in both global signals (MAP and HR) and is also detectable for SBF. In comparison to HR and SBF, MAP demonstrates higher correlation and a broader peak in the M-band, ranging up to scale values of 20–25 s.

The third and broadest peak at 34 s (corresponding pseudo-frequency 0.029 Hz in A-band) is only detectable for wavelet coherence between *m*_0_-based ΔHbO and SBF.

Wavelet coherence values obtained between all physiological signals and ΔHbO based on different moments are clearly different, with *m*_0_-based signals exhibiting the highest correlation at all scales, followed by *m*_1_-based and then by *V*-based signals. In contrast, wavelet coherence values obtained for ΔHbR, based on all three moments, are not significantly different from each other. Generally higher wavelet coherence values were observed for ΔHbO in comparison to ΔHbR for *m*_0_- and *m*_1_- based signals. Interestingly, the *V*-based ΔHbO and ΔHbR wavelet coherence values are similar at all scales, while the *m*_0_- and *m*_1_-based ΔHbO coherence is always higher than that of ΔHbR for all signals (see Figure 7).

**Figure 8:**
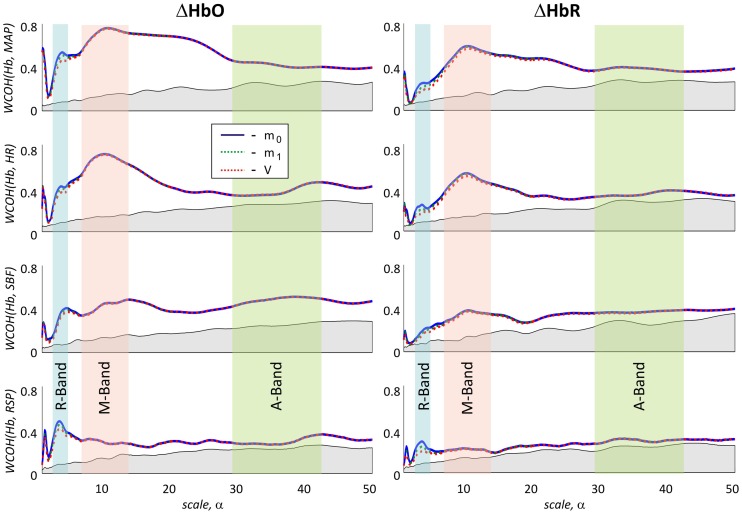
**Influence of photon noise on wavelet coherence**. The wavelet coherence between ΔHbO **(left)** or ΔHbR **(right)** signals based on different moments and four physiological signals. In the rows from **top** to **bottom** results for MAP, HR, SBF, and RSP are shown, respectively. Haemoglobin concentration changes extracted from *m*_0_, are plotted with a blue line. Green and red dashed lines correspond to experimental *m*_0_–based signals matched in the level of photon noise to *m*_1_ and *V* based signals by adding synthetic noise. R-, M-, and A-bands are marked as in Figure 4. Slight effects of matching photon noise is observed in R-band only.

The lower wavelet coherence obtained for fNIRS signals based on higher moments may be rationalized by two different explanations. The higher coherence of *m*_0_-based signals may originate from larger contributions of physiological fluctuations in the extra-cerebral skin tissue in these signals. On the other hand, the lower coherence in *m*_1_- and *V*-based signals may be due to higher photon noise levels as compared to *m*_0_-based signals. The estimation of latter effect is presented in Figure 8. It illustrates a hypothetical case assuming that the difference between signals based on different moments is only attributed to the different photon noise level. The blue solid lines show the experimentally obtained wavelet coherence for the *m*_0_-based signal. The dashed lines represent the same signal, but numerically matched to the level of photon noise present in *m*_1_- and *V*-based signals. A slight influence of photon noise is clearly visible for low scales in the R-band. In contrast, the impact of photon noise may be neglected for scales higher than 5 s (see Figure 8).

In the following we describe the time lags between physiological signals and fNIRS signals in the three main frequency bands exhibiting high impact of physiological noise. These results are presented for the R-, M-, and A-bands in Tables 1–3, respectively.

**Table 1:** **Mean group time lag between fNIRS signals and the respiration signal in the R-band**.

**R-band**		**ΔHbO, s**	**ΔHbR, s**
RSP	*m*_0_	(1.05 ± 0.33) (^*^, *T* = 11)	(0 ± 0.84)
	*m*_1_	(0.92 ± 0.3) (^*^, *T* = 11)	(0 ± 0.92)
	*V*	(0.68 ± 0.69) (^*^, *T* = 3.7)	(−0.44 ± 0.7) (^*^, *T* = 2.34)

*Asterisks indicate values significant on the group level analysis, and the T-values are listed*.

**Table 2:**
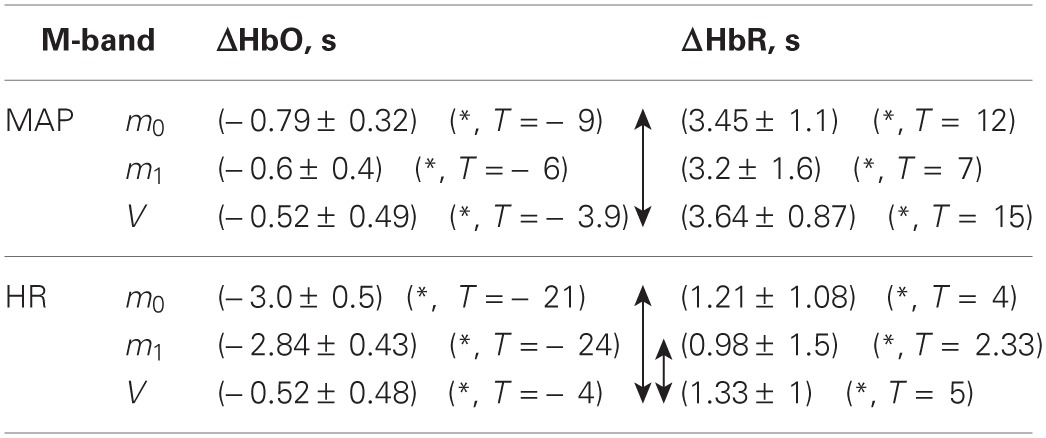
**Mean group time lag between fNIRS signals and physiological signals in M-band**.

*Asterisks indicate values significant on the group level analysis, and the T-values are listed. The arrows indicate the significant difference between parameters*.

**Table 3:** **Mean group time lag between fNIRS signals and SBF signal in A-Band**.

**A-band**		**ΔHbO, s**	**ΔHbR, s**
SBF	*m*_0_	(−6.9 ± 3.9) (^*^, *T* = −6,7)	(0 ± 8)
	*m*_1_	(0 ± 8.8)	(0 ± 6.6)
	*V*	(0 ± 10)	(−5.8 ± 7.3) (^*^, *T* = 2.95)

*Asterisks indicate values significant on the group level analysis, and the T-values are listed*.

The time delay between the respiration signal and ΔHbO was around 1 s, with no significant difference between delays obtained for ΔHbO based on different moments. The time delays for ΔHbR based on the three moments were not significantly different from zero (see Table 1).

The delay between ΔHbO and MAP was around −0.6 s and was significantly shorter for the *m*_1_- and *V*-based signals in comparison with the *m*_0_-based signal (see Table 2). The difference in time delay between *m*_0_ and *V* was (0.27 ± 0.1) s. The time delay between ΔHbR and MAP was around 3.3 s and was not significantly different for signals based on different moments.

The time delays between ΔHbO and SBF in the A-band are presented in Table 3. A significant delay of −6.0 s was observed for the *m*_0_-based ΔHbO signal.

**Figure 9:**
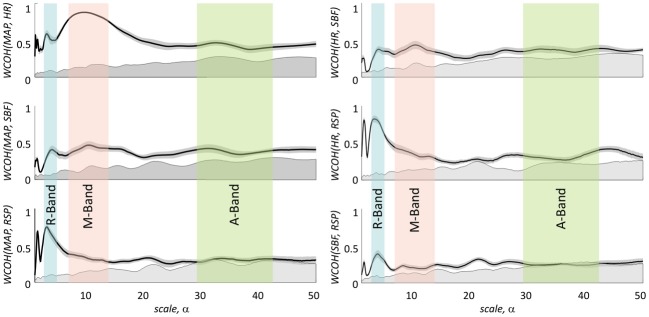
**Magnitude values of group average of wavelet coherence between four physiological signals**. Left column: in the three rows from top to bottom results for wavelet coherence between MAP and HR, SBF, and RSP are shown, respectively. Right column: in the three rows from top to bottom results for wavelet coherence between HR and SBF, HR and RSP, and SBF and RSP are shown. Light blue, light red, and light green bars indicate the following three time scale ranges: R-band (scales around 3 s and pseudo-frequencies around 0.3 Hz), M-band (scales around 10 s and pseudo-frequencies around 0.1 Hz) and A-band (scales around 35 s and pseudo-frequencies around 0.033 Hz), respectively. The gray shadowed areas around the curves represent standard error of mean for each curve.

The results of group wavelet coherence between the four physiological signals are presented in Figure 9. A maximum in wavelet coherence in R-band can be seen for all pairs of physiological signals, with highest coherence in this band observed between MAP and RSP and between HR and RSP. Very high coherence (mean value 0.85) was observed between MAP and HR signals in the M-band. Maxima of lower amplitude in the M-band were observed for coherence between MAP and SBF as well as between HR and SBF. No considerable coherence was observed between any pair of physiological signals in the A-band. Table 4 shows the group average time delays between physiological signals in R- and M-bands.

**Table 4:** **Mean group time lag between physiological signals**.

		**Time lag, s**
MAP	RSP	1 (R-band)
	HR	−2.2 (M-band)
	SBF	−0.8 (M-band)
HR	RSP	−0.2 (R-band)
	SBF	1 (M-band)
SBF	HR	0 (R-band)

### Physiological de-noising

Based on the qualitative analysis of group wavelet coherence presented in Figure 7, we identified the most relevant frequency bands and most relevant sources of physiological noise in each band. We then constructed auxiliary physiological regressors modeling impact of each process on fNIRS signals. Taking into account the mutual correlation of physiological signals that is obvious from the results presented in Figure 9, only a single physiological signal, the one showing the highest coherence, was used in each band for the de-noising procedure. In this way we avoid redundancy in our model. The RSP signals was used in order to model physiological noise in the R-band, the MAP signal was used in order to model the physiological noise component in the M-band. The physiological noise modeling in the A-band was based on the SBF signal.

In the following we summarize filter functions and time shifts employed for each physiological auxiliary regressor. This parameter choice was based on maxima in the group wavelet coherence (see Figure 7) and group mean time shifts for the corresponding bands (see Tables 1–3). To model respiration-induced noise, RSP signals were filtered with a bandpass filter [bandwidth (bw) 0.2 to 0.5 Hz] and time shifted by 1 and 0 s for ΔHbO and ΔHbR, respectively.

The impact of physiological noise at M-band frequencies was taken into account by bandpass filtering of MAP signals (bw 0.15–0.08 Hz) and a time shift of −0.69 s for ΔHbO and 3.4 s for ΔHbR signals.

To account for physiological noise in the A-band, SBF signals were band pass filtered (bw 0.02–0.04 Hz) and shifted in time by −7 s for both, ΔHbO and ΔHbR signals.

Time shifted and band-pass filtered physiological signals of each subject were normalized to unit power and used to create an individual set of four auxiliary physiological regressors for each subject. Figure 5B shows the time traces of four GLM regressors for one representative subject. GLM including one functional (cerebral) and four physiological regressors was performed for the four detector channels and two haemoglobin concentrations for each of the 14 subjects. In order to quantify the impact of physiological de-noising, we additionally performed GLM modeling with a reduced model, including the cerebral regressor only. The results of subject level analysis were subjected to a group *T*-test to determine the significance of activation at the group level.

**Figure 10:**
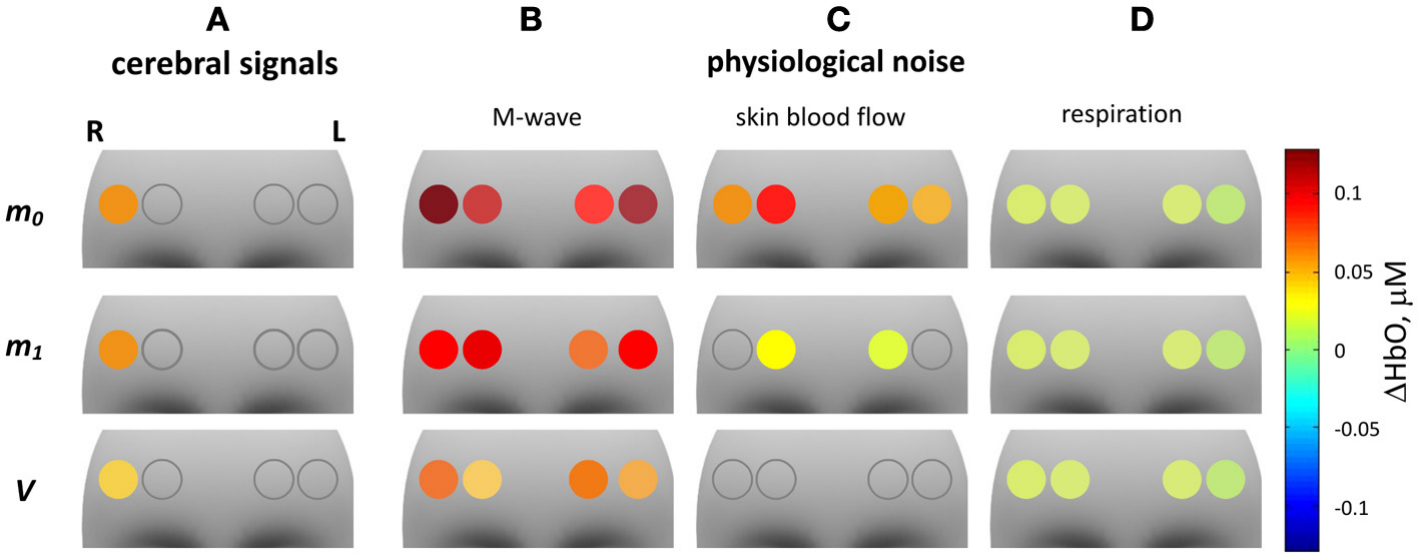
**fNIRS GLM group analysis of ΔHbO signals**. The 12 gray shaded areas indicate the forehead region. Circles indicate the positions of the four fNIRS channels. The color of the circles represents the mean of significant HbO concentration changes related to one of the four regressors in the GLM model. An empty circle indicates non-significance of the corresponding parameter. From left to right the four columns represent: HbO concentration changes related to cerebral activation and to physiological noise: **(A)** cerebral activation, **(B)** Mayer waves, **(C)** skin blood flow, **(D)** respiration. The three rows (from top to bottom) correspond to: ΔHbO based on *m*_0_, *m*_1_, and *V*.

**Figure 11:**
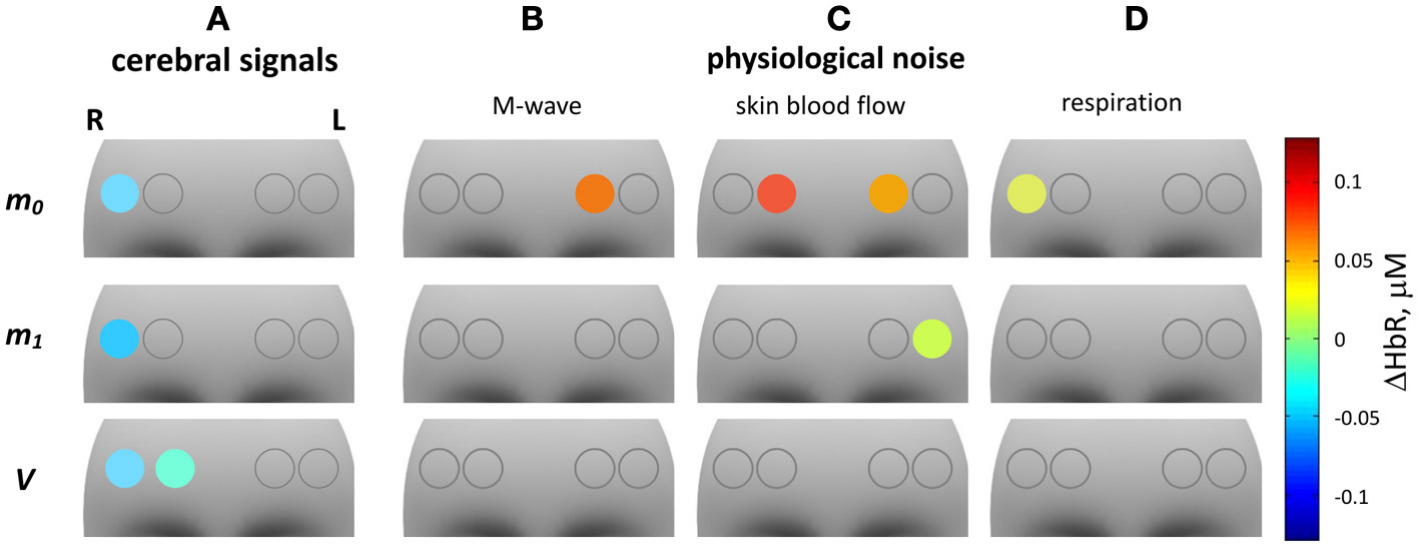
**fNIRS GLM group analysis of ΔHbR signals**. The 12 gray shaded areas indicate the forehead region. Circles indicate the positions of the four fNIRS channels. The color of the circles represents the mean of significant ΔHbR concentration changes related to one of four regressors in the GLM model. From left to right the four columns represent: ΔHbR related to cerebral activation and to physiological noise: **(A)** cerebral activation, **(B)** Mayer waves, **(C)** skin blood flow, **(D)** respiration. The three rows (from top to bottom) correspond to: ΔHbR based on *m*_0_, *m*_1_, and *V*.

The results of the second level analysis are presented in Table 5, and in Figures 10, 11 for ΔHbO and ΔHbR, respectively. In Figures 10A, 11A ΔHbO and ΔHbR attributed to cerebral activation are shown. Significant positive HbO and significant negative HbR concentration changes were observed in channel 4 for *m*_0_-, *m*_1_- as well as for *V*-based signals. For the *V*-based ΔHbR signal significant negative changes were also observed in channel 3.

**Table 5a:** **The results of second level GLM analysis of the *m*_0_-, *m*_1_- and *V*-based ΔHbO signals**.

**Process**		**Ch 1, nM**	**Ch 2, nM**	**Ch 3, nM**	**Ch 4, nM**
Cerebral activation	*m*_0_	–	–	–	(50 ± 67)
					(*T*= 2.8)
	*m*_1_				(69 ± 111)
					(*T* = 2.4)
	*V*				(40 ± 53)
					(*T* = 2.84)
M-wave	*m*_0_	(94 ± 49)	(74 ± 46)	(79 ± 57)	(107 ± 55)
		(*T* = 7.0)	(*T* = 6.0)	(*T* = 5.2)	(*T* = 7.4)
	*m*_1_	(78 ± 41)	(54 ± 31)	(81 ± 46)	(83 ± 43)
		(*T* = 7.1)	(*T* = 6.4)	(*T* = 6.6)	(*T* = 7.1)
	*V*	(57 ± 30)	(38 ± 20)	(55 ± 31)	(57 ± 36)
		(*T* = 7.2)	(*T* = 7.1)	(*T* = 6.7)	(*T* = 5.97)
Skin blood flow	*m*_0_	(47 ± 54)	(52 ± 33)	(81 ± 74)	(57 ± 75)
		(*T* = 3.3)	(*T* = 5.8)	(*T* = 4.0)	(*T* = 2.8)
	*m*_1_	–	(16.8 ± 23.5)	(24.4 ± 23.1)	–
			(*T* = 2.7)	(*T* = 3.96)	
	*V*	–	–	–	–
Respiration	*m*_0_	(8 ± 10)	(10 ± 10)	(11 ± 10)	(10 ± 9)
		(*T* =2.9)	(*T* = 3.6)	(*T* = 4.3)	(*T* = 4.9)
	*m*_1_	(9 ± 10)	(7 ± 7)	(10 ± 9)	(9 ± 10)
		(*T* = 3.5)	(*T* =3.7)	(*T* =3.8)	(*T* =3.42)
	*V*	(6 ± 6.5)	(6 ± 6.5)	(5 ± 5.8)	–
		(*T* = 3.19)	(*T* = 3.32)	(*T* = 2.95)	

*Only values significant on the group level analysis are shown, and the T-values are listed*.

**Table 5b:** **The results of second level GLM analysis of the *m*_0_-, *m*_1_,- and *V*-based ΔHbR signals**.

**Process**		**Ch 1, nM**	**Ch 2, nM**	**Ch 3, nM**	**Ch 4, nM**
Cerebral activation	*m*_0_	–	–		(−35 ± 36)
					(*T* = −3.5)
	*m*_1_			–	(−44 ± 43)
					(*T* = −3.6)
	*V*			(22 ± 35)	(−34 ± 32)
				(*T* = −2.2)	(*T* = −3.9)
M-wave	*m*_0_	–	(54 ± 57)	–	–
			(*T* = 3.5)		
Skin blood flow	*m*_0_	–	(60 ± 97)	(50 ± 55)	–
			(*T* = 2.3)	(*T* = 3.4)	
	*m*_1_	–	–	–	(10 ± 13)
					(*T* = 2.7)
Respiration	*m*_0_	–	–	–	(11 ± 12)
					(*T* = 2.2)

*Only values significant on the group level analysis are shown, and the T-values are listed*.

Subpanels B–D in Figures 10, 11 show significant ΔHbO and ΔHbR values attributed to the three components of the physiological noise related to M-wave, SBF and respiration. One can see that the impact of physiological noise is generally much stronger for ΔHbO than for ΔHbR. Noise related to M-wave and respiration is present in ΔHbO based on all three moments, while essentially only *m*_0_-based ΔHbR is affected. The physiological noise related to the SBF changes is present only in *m*_0_ and *m*_1_-based signals and is stronger pronounced in the two medial channels. In the *V*-based ΔHbR no significant contribution of physiological noise was detected.

The results of the reduced GLM including only one cerebral regressor are presented in Table 6. No significant cerebral activation was observed with this reduced model for *m*_0_-based ΔHbO. Significant activation was identified for channel 4 only, for the *m*_1_- and *V*-based ΔHbO signals. In ΔHbR signals significant activation was observed in channel 4 for the signals based on all three moments, however, the obtained *T*-values were always lower than that observed with the full model (compare Table 5, 6).

**Table 6:** **The results of the reduced GLM model including only cerebral regressor**.

**HbO**		**Ch 1, Nm**	**Ch 2, Nm**	**Ch 3, Nm**	**Ch 4**
Cerebral activation	*m*_0_	–	–	–	–
	*m*_1_				(75 ± 129)
					(*T* = 2.2)
	*V*				(50 ± 81)
					(*T* = 2.3)
**HbR**		**Ch 1, nM**	**Ch 2, nM**	**Ch 3, nM**	**Ch 4, nM**
Cerebral activation	*m*_0_	–	–	–	(−40 ± 50)
					(*T* = −3.5)
	*m*_1_				(−36 ± 41)
					(*T* = −3.3)
	*V*				(−45 ± 47)
					(*T* = −3.6)

## Discussion

The wavelet coherences of fNIRS signals and physiological processes, presented in Figures 7, 9, reveal a strong impact of the physiological noise at three main frequency bands: R-band with scales around 3 s (0.3 Hz), M-band with scales around 10 s (0.1 Hz) and A-band with scales around 34 s (0.034 Hz). These three distinct bands indicate the presence of at least three distinct physiological mechanisms dominating physiological fNIRS noise. In the following we summarize the results obtained at each band and discuss possible underlying physiological mechanisms.

### R-band (0.2 to 0.5Hz)

The R-band correlation peak is most obvious for the wavelet coherence between *m*_0_-based ΔHbO signal and the respiratory signal (see Figures 7, 10D). Its peak frequency (0.3 Hz) corresponds to the mean respiration rate, thus indicating the direct influence of the respiration on the fNIRS signal. Clear coherence at the R-band was also found for *m*_0_-based MAP, HR and SBF signals for both ΔHbO and ΔHbR concentration changes. However, the peak is not always clearly detectable, due to an overlay from neighboring M-band maxima in these signals.

Coherence at R-band demonstrates a clear difference between signals based on different moments. Highest coherence was primarily observed for *m*_0_-based signals. Significantly lower values were observed for *m*_1_-based ΔHbO and ΔHbR signals. The coherence in the R-band for *V*-based signal does not exceed the noise level. However, this dependence on the moment, which at first glance seems to be related to different physiological noise levels at different depths, has to be interpreted with care.

The analysis of the photon noise influence presented in Figure 8 indicates that the wavelet coherence at small scale values is sensitive to photon noise. Taking into account an increasing photon noise contribution in *m*_1_- and *V*-based fNIRS signals, the observed dependence might be partly due to an artifact induced by different photon noise levels in the three measurements based on different moments.

In literature two different physiological mechanisms leading to R-band fluctuations are described. First, there is a direct influence of the respiration on the venous blood flow. Negative intrathoracic pressure during inspiration leads to increased venous outflow, which modulates the venous blood volume with the respiration frequency. The second mechanism is the respiratory modulation of the HR, mediated by the parasympathetic nervous system. Consequently the arterial input is modulated via HR by the respiratory cycle, a phenomenon usually referred to as respiratory sinus arrhythmia (Hirsch and Bishop, 1981). High correlation between respiration and HR observed in our study (see Figure 9) also indicates the presence of this phenomenon. Based on our data it is difficult to conclude, which of the two mechanisms dominates fNIRS physiological noise—direct influence of the respiratory pump, or the indirect influence of the HR variability. It is possible that the impact of both mechanisms is different for the two haemoglobin concentrations.

### M-band (0.05 to 0.15Hz)

The highest wavelet coherence between fNIRS signals and MAP and HR traces was observed in the M-band. This coherence is induced by Mayer waves, correlated fluctuation between HR and MAP in this frequency band (Elstad et al., 2011). As one can see in Figure 7, Mayer waves are present in both ΔHbO and ΔHbR concentration changes. However, both haemoglobin concentrations demonstrate different amplitudes, different time shifts and different dependence on moment, indicating different depth localization in the tissue. As one can see from the results of the WCA presented in Figure 7 and from GLM results in Figure 10, *m*_0_-based ΔHbO signals show the highest M-band contribution. The amplitude of M-band physiological noise is higher than that of the cerebral signal. ΔHbO signals based on *m*_1_ and *V* show significantly lower Mayer wave contributions. Since the influence of photon noise is negligible in the M-band, this moment dependence can be clearly assigned to Mayer waves with different amplitudes at different depths. A large fraction of the M-band contribution in the ΔHbO signal originates from superficial extra-cerebral tissue, however, there is an additional cerebral contribution as well. The presence of two separate M-band components (extra- and intracranial) is further supported by the phase difference detected between *m*_0_-, *m*_1_-, and *V*-based signals and reported in Table 2. Indeed, if the Mayer waves appear both in the brain and in the scalp, one might expect a time delay between these two compartments, caused by different vascular path lengths as well as possible delays in sympathetic mediating signals between the two compartments (Tong et al., 2011). Although cerebral auto-regulation mostly acts at lower frequencies (Latka et al., 2005; Rowley et al., 2007), there might be a time shift due to this vascular property of brain vessels as well. Since *m*_0_-, *m*_1_-, and *V*-based signals reflect a linear combination of signals from skin and cerebral compartments with different weights, they would then show different time lags relative to MAP.

Interestingly, wavelet coherence between ΔHbR and MAP and between ΔHbR and HR is very similar for *m*_0_, *m*_1_, and *V*-based signals. In addition, no significant time lag was obtained for these signals. This means that the main source of M-band physiological noise in ΔHbR is localized in deeper intracranial tissue. However, GLM analysis failed to detect significant ΔHbR M-band contribution in *V*-based signals (see Figure 11), although significant M-band signals were obtained in *m*_1_ and *V*-based ΔHbO signals. Therefore, we can conclude that both extra- and intracranial M-band signals are mostly present on arterial side. This fact might explain why Mayer waves are not considered as important source of physiological noise in fMRI (Birn et al., 2006; Chang and Glover, 2009). Since BOLD signal exploited in fMRI mostly reflects changes in HbR concentration, it seems to be not strongly affected by Mayer waves. Moreover, the cerebral compartment might exhibit an additional contribution in the M-wave band which is not coupled with blood pressure fluctuations as demonstrated recently by (Rayshubskiy et al., 2013). The presence of an additional component that is uncorrelated with MAP could also explain the experimentally observed lower wavelet coherence in the deeper tissue.

Another interesting fact is the high coherence between fNIRS signals and HR time traces in the M-band. Although the direct correlation between HR and signals is low, it accounts for up to 40% fNIRS signal variance for conventional cw (in our case *m*_0_-based) signals, for HR being shifted in time. Despite this number is lower than that obtained for MAP, it is still enough to significantly improve fNIRS sensitivity when used in a de-noising procedure. This may become a fact of high importance in fNIRS experimental practice. Indeed, continuous blood pressure measurements are challenging and dedicated hardware more rare and cost intensive than conventional ECG or pulse plethysmography devices. Those are relatively cheap and available in many labs. Thus, physiological de-noising based on HR measurements is much more easily applicable than approaches based on continuous MAP recordings, although it might be slightly less efficient.

High correlation between MAP, HR, and fNIRS signals was reported and emphasized by several studies (Franceschini et al., 2006; Katura et al., 2006; Minati et al., 2011; Li et al., 2013). The time shift between fNIRS signals and MAP and HbO signals reported by (Katura et al., 2006) are very close to those presented in the Table 2.

Finally we would like to discuss the possible physiological mechanism inducing Mayer waves in fNIRS signals. Synchronized oscillations with frequencies around 0.1 Hz are typically observed in blood pressure and HR in humans and animals and are most likely induced by an interplay between sympathetically driven HR variations and sympathetic vasoconstriction of peripheral resistive vessels (Pagani et al., 1986; Malliani et al., 1991; Stauss et al., 1998; Cohen and Taylor, 2002; Nilsson and Aalkjaer, 2003). The high coherence between MAP and HR in M-band observed in our experiment (see Figure 9) is in agreement with these findings.

A mechanism that directly links local blood pressure and local HbO and HbR concentration changes is vasoconstriction of peripheral resistance vessels. These vessels are situated prior to the capillary bed on the arterial side and therefore reveal high concentration of oxygenated haemoglobin. The diameter of resistive vessels is sympathetically regulated, and critically influences the overall hydrodynamic resistance of the vascular system and thereby the blood pressure. Regulatory changes in the vessel diameter are connected to changes of the arterial blood volume, which is the most probable source of the observed tissue haemoglobin concentration changes. These changes occur on the arterial side are further propagated to the venous side due to induced fluctuations in blood flow (Tong et al., 2011).

### A-band (0.02 to 0.04Hz)

As one can see in Figure 7 the WCA reveals synchronous oscillations in the very low frequency band between the *m*_0_-based ΔHbO signal and SBF.

With the help of GLM analysis presented in Figures 10, 11 we detect a significant contribution of this signal in all four channels in *m*_0_-based ΔHbO, and in the two medial channels in *m*_1_-based ΔHbO and *m*_0_-based ΔHbR and in one lateral channel in *m*_1_-based ΔHbR. Since *m*_0_ is much more sensitive to superficial tissue then *m*_1_ and *V*, we can conclude that activation in the A-band is predominantly localized in the skin compartment, and is due to the local SBF regulation mechanisms.

The stronger influence of the SBF related artifact on the medial forehead is supported by our earlier findings from a comparative fMRI/ fNIRS study (Kirilina et al., 2012). In this study we observed a task-evoked response in the two medial veins draining the forehead.

The scale of maximum coherence corresponds to the period of stimulation (34 s) used in our study. Therefore, we hypothesize, that observed SBF responses might be induced by task-induced cognitive stress.

VLFOs with frequencies from 0.021 to 0.052 Hz were previously reported in LDF and NIRS measurements of the skin (Li et al., 2010) and were linked to sympathetic control of the peripheral vasculature (Kastrup et al., 1989; Söderström et al., 2003). Since increased level of sympathetic activity might be induced by cognitive or emotional tasks used in fNIRS experiments, SBF oscillation in this band might be synchronized with the task. Therefore, special caution has to be taken in fNIRS signal analysis in order to separate these superficial skin signals from cerebral activation.

### Physiological de-noising of fNIRS data

The results of GLM-modeling presented in Figures 10A, 11A demonstrate significant activation in the lateral left channel. An increase in HbO and decrease in HbR concentration was observed in this area when physiological de-noising was applied. This result is consistent with the results of an fMRI study performed using the same task and the same subject group (Kirilina et al., 2012). In this study activation in bilateral Brodman Area 10 was observed, lateralized on the right side (Talairach coordinates [32 48 11]). The left BA10 activation was too deep from the cortical surface to be detected by fNIRS.

Although ΔHbO demonstrates the signals of higher absolute value than that of ΔHbR, it is also much more strongly affected by physiological noise (compare Figures 10B–D, 11B–D). Moreover, for the *m*_0_-based signal we failed to detect cerebral activation in ΔHbO when the reduced GLM model (Table 6) was used. This means that we would not detect any cerebral activation with conventional cw fNIRS, if not corrected for physiological noise. In contrast, ΔHbR signals show robust activation in all three moments even without physiological noise correction (see Figure 11).

The above described results demonstrate that ΔHbR signals are more reliable in detecting cerebral activation, and that the de-noising procedure developed in the current study can significantly improve the sensitivity of fNIRS to cerebral activation for ΔHbO.

### Limitations of the current study and outlook

One limitation of the present approach is that not all possible contributions to fNIRS physiological noise might be detected by our approach, but only those, which also manifest themselves in MAP, HR, and SBF. In particular, Lased Doppler Flowmetry measures only capillary SBF and not that of the large vessels. We also did not account for the possible impact of fluctuations in arterial CO_2_ concentration (Scholkmann et al., 2013a,b). Thus, some important sources of signal variance might be missing in our consideration.

Moreover the wavelet analysis is a linear transformation method, thus with the present approach we might miss more complex interrelations between physiological parameters and fNIRS signals, which are not captured by a simple linear relationship. In our WCA we used single physiological trace at a time. In the future the multi-variable coherence and correlation analysis such as the canonical correlation analysis could provide more complete picture (Caicedo et al., 2013).

Another limitation of the proposed de-noising strategy is the necessity to use additional physiological sensors and monitors. In particular, continuous monitoring of the blood pressure as well as laser flowmetry are not widely available in most labs. However, we have shown that even applying widely available and cost efficient sensors for respiration and HR might significantly improve fNIRS data quality.

An important interesting step in the further investigation might be to compare and combine the presented method of physiological de-noising with methods based on anatomical localization of cerebral and extra-cerebral tissue such as superficial signal regression.

## Conclusion

In the current study we investigated the impact of global systemic and local regulatory physiological processes on physiological noise in fNIRS measurements and developed a method for physiological de-noising of fNIRS data. Global systemic processes were quantified by measuring MAP, HR, and respiration. Local regulatory processes in the skin were measured by means of SBF recordings. WCA was employed to characterize the contribution of these physiological parameters on fNIRS noise, at different time scales. Time-domain measurements in combination with signals, based on different moments of DTOF, enabled us to obtain information on the depth localization of different physiological noise sources.

With the help of these methods we were able to identify three main mechanisms contributing to physiological noise in fNIRS signals at three time scales. Two of these processes were induced by global systemic physiology: Mayer waves and respiration. The third slow process was induced by local blood flow changes in the skin tissue. We show that HbO signals are more strongly affected by global processes in both, extra- and intra-cerebral compartments, and local SBF regulation, while HbR signals are less contaminated by extra-cerebral processes.

By means of GLM analysis and auxiliary physiological regressors we quantified the relative impact of each process. Moreover, we propose a de-noising algorithm and demonstrate its performance on a functional experiment on the forehead. The proposed method was shown to significantly improve the sensitivity of fNIRS to cerebral activation.

### Conflict of interest statement

The authors declare that the research was conducted in the absence of any commercial or financial relationships that could be construed as a potential conflict of interest.

## Appendix

The concentration changes of HbO and HbR were determined based on three moments of DTOFs (*m*_0_, *m*_1_, and *V*) separately. The time traces of ΔHbO and ΔHbR related to the different moments exhibit different levels of non-physiological noise, due to the different influence of instrumental noise. In order to estimate the amplitude of this non-physiological noise in ΔHbO and ΔHbR time traces, in the first step we estimated the instrumental and photon noise induced standard deviation of the corresponding moments. In the second step, we investigated how this uncertainty is propagated through the calculation of ΔHbO and ΔHbR.

**Step 1**. It is assumed that the uncertainty of measurement is dominated by photon noise following a Poisson distribution. For this case the variances of the changes in moments are determined by (Liebert et al., 2003, 2012):

(A1) $$\sigma^{2}\left(\Delta M_{0}\left(\lambda_{i}\right)\right)\approx \sigma^{2}\left(\Delta m_{0}\right)/m_{0}^{2}\approx 1/m_{0}\left(\lambda_{i}\right)$$

(A2) $$\sigma^{2}\left(\Delta M_{1}\left(\lambda_{i}\right)\right)\approx M_{2,\text{raw}}\left(\lambda_{i}\right)/m_{0}\left(\lambda_{i}\right)$$

(A3) $$\sigma^{2}\left(\Delta M_{2}\left(\lambda_{i}\right)\right)\approx \left(M_{4,\text{ raw}}\left(\lambda_{i}\right)-M_{2,\text{raw}}^{2}\left(\lambda_{i}\right)\right)/m_{0}\left(\lambda_{i}\right)$$

where Δ*M*_0_ = −ln(1 − Δ*m*_0_/*m*_0_) is the attenuation change, Δ*M*_1_ = Δ*m*_1_, Δ*M*_2_ = Δ*V*. *M*_2, raw_ = *V*_raw_ and *M*_4, raw_ are the variance (second central moment) and the fourth central moment of the DTOF, respectively, as directly obtained in the experiment, without eliminating the influence of the instrumental response function. *m*_0_, *M*_2, raw_ and *M*_4, raw_ were estimated from the experimentally measured DTOFs for each subject and each wavelength. Based on the group-averaged values of these parameters we estimated the standard deviations of each moment according to Equations (A1)–(A3) (see Table A1).

**Table A1:** **Group-averaged values of sensitivity factors *S*_*n*_ and of standard deviations due to photon noise σ(Δ*M*_*n*_) of the changes in moments for all three wavelengths**.

**Wavelength λ_*i*_**	**689 nm**	**797 nm**	**828 nm**
*S*_0_/*cm*	19.2	17.91	17.1
σ(Δ*M*_0_)	0.004	0.005	0.0054
*S*_1_/(*cm ns*)	−2.7	−2.6	−2.4
σ(Δ*M*_1_)/*ps*	2	2.3	2.6
*S*_2_/(*cm ns*^2^)	−1.21	−1.3	−1.3
σ(Δ*M*_2_)/(10^−3^*ns*^2^)	1.8	1.9	2.3

**Step 2**. In order to obtain the concentration changes of oxy- and deoxyhaemoglobin, Δ*HbO*_*n*_ and Δ*HbR*_*n*_, based on the *n*-th moment we need to solve the over-determined set of linear equations:

(A4) $$\left(\begin{array}{c} \begin{array}{l} \Delta M_{n}\left(\lambda_{1}\right)\\ \Delta M_{n}\left(\lambda_{2}\right)\\ \Delta M_{n}\left(\lambda_{3}\right) \end{array} \end{array}\right)\text{​}=\text{​}\ln(10)\left[\begin{array}{c} \begin{array}{l} S_{n}\left(\lambda_{1}\right)\epsilon_{\text{HbO}}\left(\lambda_{1}\right)S_{n}\left(\lambda_{1}\right)\epsilon_{\text{HbR}}\left(\lambda_{1}\right)\\ S_{n}\left(\lambda_{2}\right)\epsilon_{\text{HbO}}\left(\lambda_{2}\right)S_{n}\left(\lambda_{2}\right)\epsilon_{\text{HbR}}\left(\lambda_{2}\right)\\ S_{n}\left(\lambda_{3}\right)\epsilon_{\text{HbO}}\left(\lambda_{3}\right)S_{n}\left(\lambda_{3}\right)\epsilon_{\text{HbR}}\left(\lambda_{3}\right) \end{array} \end{array}\right]\left(\begin{array}{c} \begin{array}{l} \Delta {\text{HbO}}_{n}\\ \Delta {\text{HbR}}_{n} \end{array} \end{array}\right)$$

where Δ*M*_n_(λ_*i*_) represents the changes in the *n*-th moment (*n* = 0, 1, and 2) (definitions see above) for the *i*-th wavelength. *S*_*n*_(λ_*i*_) is a moment-specific sensitivity factor, ϵ_HbO_(λ_*i*_) and ϵ_HbR_(λ_*i*_) are the molar absorption coefficients of HbO and HbR, The sensitivity factors (for the homogeneous case) can be derived from moments of the measured DTOF by (Arridge et al., 1992; Steinbrink, 2000; Liebert et al., 2004):

(A5) $$S_{0}\left(\lambda_{i}\right)=c_{m}M_{1}\left(\lambda_{i}\right)$$

(A6) $$S_{1}\left(\lambda_{i}\right)=-c_{m}M_{2}\left(\lambda_{i}\right)$$

(A7) $$S_{2}\left(\lambda_{i}\right)=-c_{m}M_{3}\left(\lambda_{i}\right)$$

where *M*_1_ = *m*_1_, *M*_2_ = *V*, *M*_3_—third central moment of the DTOF and *c*_*m*_—speed of light in the medium. Note that *S*_0_ is related to the differential pathlength factor (*D*_*PF*_) by *S*_0_ = *D*_*PF*_*r*_*sd*_ with *r*_*sd*_ being the source-detector separation.

Equation (A4) can be rewritten as $\vec{\Delta M_{n}}=A_{n}\vec{\Delta C_{n}}$ where $\Delta C_{n}=\left(\begin{array}{l} \Delta {\text{HbO}}_{n}\\ \Delta {\text{HbR}}_{n} \end{array}\right)$ is the vector of concentration changes, $\vec{\Delta M_{n}}$ the vector of changes of the *n*−th moment for the three wavelengths and *A_n_* the coefficient matrix on the right-hand side of Equation (A4). The solution of this system of equations in a least-squares sense is given by:

(A8) $$\vec{\Delta C_{n}}={\left(A_{n}^{T}A_{n}\right)}^{-1}A_{n}^{T}\vec{\Delta M_{n}}$$

The variances (i.e., squares of standard deviations) of the concentration changes ΔHbO_n_ and ΔHbR_n_ are the diagonal elements of the covariance matrix:

(A9) $$\text{cov}\left(\vec{\Delta C_{n}},\vec{\Delta C_{n}}\right)={\left(A_{n}^{T}Z_{n}{}^{-1}A_{n}\right)}^{-1}$$

Herein *Z_n_* is the covariance matrix of the moment changes $\vec{\Delta M_{n}}$. Since photon noise is uncorrelated for the measurements at different wavelengths, *Z_n_* is a diagonal matrix:

(A10) $$\begin{array}{l} Z_{n}=\text{cov}\left(\vec{\Delta M_{n}},\vec{\Delta M_{n}}\right)\\ \;=\left[\begin{array}{ccc} \sigma^{2}\left(\Delta M_{n}\left(\lambda_{1}\right)\right) & 0 & 0\\ 0 & \sigma^{2}\left(\Delta M_{n}\left(\lambda_{2}\right)\right) & 0\\ 0 & 0 & \sigma^{2}\left(\Delta M_{n}\left(\lambda_{3}\right)\right) \end{array}\right] \end{array}$$

with σ (Δ*M_n_*(λ_*i*_) being the standard deviation of the *n*-th moment at wavelength λ_*i*_.

For the estimation of the standard deviations of the concentration changes we used the group-averaged values of the sensitivity factors together with the group-averaged standard deviations of moments obtained in Step 1, both listed in Table A1.

## References

[B1] Aletti F. Re R. Pace V. Contini D. Molteni E. Cerutti S. (2012). Deep and surface hemodynamic signal from functional time resolved transcranial near infrared spectroscopy compared to skin flowmotion. Comput. Biol. Med. 42, 282–289 10.1016/j.compbiomed.2011.06.001 21742320

[B2] Arridge S. R. Cope M. Delpy D. T. (1992). The theoretical basis for the determination of optical pathlengths in tissue: temporal and frequency analysis. Phys. Med. Biol. 37, 1531 10.1088/0031-9155/37/7/005 1631197

[B3] Birn R. M. Diamond J. B. Smith M. A. Bandettini P. A. (2006). Separating respiratory-variation-related fluctuations from neuronal-activity-related fluctuations in fMRI. Neuroimage 31, 1536–1548 10.1016/j.neuroimage.2006.02.048 16632379

[B4] Caicedo A. Papademetriou M. D. Elwell C. E. Hoskote A. Elliott M. J. Van Huffel S. (2013). Canonical correlation analysis in the study of cerebral and peripheral haemodynamics interrelations with systemic variables in neonates supported on ECMO. Adv. Exp. Med. Biol. 765, 23–29 10.1007/978-1-4614-4989-8_4 22879010 PMC4038010

[B5] Chang C. Glover G. H. (2009). Effects of model-based physiological noise correction on default mode network anti-correlations and correlations. Neuroimage 47, 1448–1459 10.1016/j.neuroimage.2009.05.012 19446646 PMC2995588

[B5a] Cohen M. A. Taylor J. A. (2002). Short-term cardiovascular oscillations in man: measuring and modelling the physiologies. J. Physiol. 542, 669–683 10.1113/jphysiol.2002.017483 12154170 PMC2290446

[B6] Drummond P. (1996). Adrenergic receptors in the forehead microcirculation. Clin. Auton. Res. 6, 23–27 10.1007/BF02291402 8924752

[B7] Drummond P. (1997). The effect of adrenergic blockade on blushing and facial flushing. Psychophysiology 34, 163–168 10.1111/j.1469-8986.1997.tb02127.x 9090265

[B8] Elstad M. WallØe L. Chon K. H. Toska K. (2011). Low-frequency fluctuations in heart rate, cardiac output and mean arterial pressure in humans: what are the physiological relationships? J. Hypertens. 29, 1327–1336 10.1097/HJH.0b013e328347a17a 21558953

[B9] Elwell C. E. Springett R. Hillman E. Delpy D. T. (1999). Oscillations in cerebral haemodynamics. Implications for functional activation studies. Adv. Exp. Med. Biol. 471, 57–65 10.1007/978-1-4615-4717-4_8 10659132

[B10] Franceschini M. A. Joseph D. K. Huppert T. J. Diamond S. G. Boas D. A. (2006). Diffuse optical imaging of the whole head. J. Biomed. Opt. 11:054007 10.1117/1.2363365 17092156 PMC2637816

[B11] Friston K. Stephan K. (2007). Chapter 3—modelling brain responses, in Statistical Parametric Mapping, eds Friston K. Ashburner J. Kiebel S. Nichols T. Penny W. (London: Academic Press), 32–45

[B12] Funane T. Atsumori H. Katura T. Obata A. N. Sato H. Tanikawa Y. (2013). Quantitative evaluation of deep and shallow tissue layers' contribution to fNIRS signal using multi-distance optodes and independent component analysis. Neuroimage. [Epub ahead of print]. 10.1016/j.neuroimage.2013.02.026 23439443

[B13] Gagnon L. Cooper R. J. Yücel M. A. Perdue K. L. Greve D. N. Boas D. A. (2012). Short separation channel location impacts the performance of short channel regression in NIRS. Neuroimage 59, 2518–2528 10.1016/j.neuroimage.2011.08.095 21945793 PMC3254723

[B14] Goupillaud P. Grossmann A. Morlet J. (1984). Cycle-octave and related transforms in seismic signal analysis. Geoexploration 23, 85–102 10.1016/0016-7142(84)90025-5

[B15] Gregg N. M. White B. R. Zeff B. W. Berger A. J. Culver J. P. (2010). Brain specificity of diffuse optical imaging: improvements from superficial signal regression and tomography. Front. Neuroenergetics 2:14 10.3389/fnene.2010.00014 20725524 PMC2914577

[B16] Grinsted A. Moore J. C. Jevrejeva S. (2004). Application of the cross wavelet transform and wavelet coherence to geophysical time series. Nonlin Process. Geophys 11, 561–566 10.5194/npg-11-561-2004

[B17] Hirsch J. A. Bishop B. (1981). Respiratory sinus arrhythmia in humans: how breathing pattern modulates heart rate. Am. J. Physiol. 241, H620–H629 7315987 10.1152/ajpheart.1981.241.4.H620

[B18] Hudetz A. G. Roman R. J. Harder D. R. (1992). Spontaneous flow oscillations in the cerebral cortex during acute changes in mean arterial pressure. J. Cereb. Blood Flow Metab. 12, 491–499 10.1038/jcbfm.1992.67 1569142

[B19] Johansson B. Bohr D. F. (1966). Rhythmic activity in smooth muscle from small subcutaneous arteries. Am. J. Physiol. 210, 801–806 4286041 10.1152/ajplegacy.1966.210.4.801

[B20] Julien C. (2006). The enigma of Mayer waves: facts and models. Cardiovasc. Res. 70, 12–21 10.1016/j.cardiores.2005.11.008 16360130

[B21] Kastrup J. Bülow J. Lassen N. A. (1989). Vasomotion in human skin before and after local heating recorded with laser Doppler flowmetry. A method for induction of vasomotion. Int. J. Microcirc. Clin. Exp. 8, 205–215 2659545

[B22] Katura T. Sato H. Fuchino Y. Yoshida T. Atsumori H. Kiguchi M. (2008). Extracting task-related activation components from optical topography measurement using independent components analysis. J. Biomed. Opt. 13:054008 10.1117/1.2981829 19021388

[B23] Katura T. Tanaka N. Obata A. Sato H. Maki A. (2006). Quantitative evaluation of interrelations between spontaneous low-frequency oscillations in cerebral hemodynamics and systemic cardiovascular dynamics. Neuroimage 31, 1592–1600 10.1016/j.neuroimage.2006.02.010 16549367

[B24] Kiebel S. J. Holmes A. P. (2007). Chapter 8—the general linear model, in Statistical Parametric Mapping, eds Friston K. Ashburner J. Kiebel S. Nichols T. Penny W. (London: Academic Press), 101–125

[B25] Kirilina E. Jelzow A. Heine A. Niessing M. Wabnitz H. Brühl R. (2012). The physiological origin of task-evoked systemic artefacts in functional near infrared spectroscopy. Neuroimage 61, 70–81 10.1016/j.neuroimage.2012.02.074 22426347 PMC3348501

[B26] Kohno S. Miyai I. Seiyama A. Oda I. Ishikawa A. Tsuneishi S. (2007). Removal of the skin blood flow artifact in functional near-infrared spectroscopic imaging data through independent component analysis. J. Biomed. Opt. 12:062111 10.1117/1.2814249 18163814

[B27] Latka M. Turalska M. Glaubic-Latka M. Kolodziej W. Latka D. West B. J. (2005). Phase dynamics in cerebral autoregulation. Am. J. Physiol. 289, H2272–H2279 10.1152/ajpheart.01307.2004 16024579

[B28] Li Z. Wang Y. Li Y. Wang Y. Li J. Zhang L. (2010). Wavelet analysis of cerebral oxygenation signal measured by near infrared spectroscopy in subjects with cerebral infarction. Microvasc. Res. 80, 142–147 10.1016/j.mvr.2010.02.004 20156461

[B29] Li Z. Zhang M. Xin Q. Luo S. Cui R. Zhou W. (2013). Age-related changes in spontaneous oscillations assessed by wavelet transform of cerebral oxygenation and arterial blood pressure signals. J. Cereb. Blood Flow Metab. 33, 692–699 10.1038/jcbfm.2013.4 23361392 PMC3652694

[B30] Liebert A. Wabnitz H. Elster C. (2012). Determination of absorption changes from moments of distributions of times of flight of photons: optimization of measurement conditions for a two-layered tissue model. J. Biomed. Opt. 17:057005 10.1117/1.JBO.17.5.057005 22612144

[B31] Liebert A. Wabnitz H. Grosenick D. Möller M. Macdonald R. Rinneberg H. (2003). Evaluation of optical properties of highly scattering media by moments of distributions of times of flight of photons. Appl. Opt. 42, 5785–5792 10.1364/AO.42.005785 14528944

[B32] Liebert A. Wabnitz H. Steinbrink J. Obrig H. Möller M. Macdonald R. (2004). Time-resolved multidistance near-infrared spectroscopy of the adult head: intracerebral and extracerebral absorption changes from moments of distribution of times of flight of photons. Appl. Opt. 43, 3037–3047 10.1364/AO.43.003037 15176190

[B32a] Malliani A. Pagani M. Lombardi F. Cerutti S. (1991). Cardiovascular neural regulation explored in the frequency domain. Circulation 84, 482–492 10.1161/01.CIR.84.2.482 1860193

[B33] Mallat S. G. (2009). A Wavelet Tour of Signal Processing the Sparse Way. Amsterdam; Boston, MA: Elsevier, Academic Press

[B34] Mayer S. (1876). Studien zur Physiologie des Herzens und der Blutgefässe 6. Abhandlung: Über spontane Blutdruckschwankungen. Sitzungsberichte Akad. Wiss. Wien Math.-Naturwissenschaftliche Cl. Anat. 74, 281–307

[B35] Mayhew J. E. Askew S. Zheng Y. Porrill J. Westby G. W. Redgrave P. (1996). Cerebral vasomotion: a 0.1-Hz oscillation in reflected light imaging of neural activity. Neuroimage 4, 183–193 10.1006/nimg.1996.0069 9345508

[B36] Minati L. Kress I. U. Visani E. Medford N. Critchley H. D. (2011). Intra- and extra-cranial effects of transient blood pressure changes on brain near-infrared spectroscopy (NIRS) measurements. J. Neurosci. Methods 197, 283–288 10.1016/j.jneumeth.2011.02.029 21392529 PMC3089735

[B37] Mitsis G. D. Poulin M. J. Robbins P. A. Marmarelis V. Z. (2004). Nonlinear modeling of the dynamic effects of arterial pressure and CO2 variations on cerebral blood flow in healthy humans. IEEE Trans. Biomed. Eng. 51, 1932–1943 10.1109/TBME.2004.834272 15536895

[B37a] Nilsson H. Aalkjaer C. (2003). Vasomotion: mechanisms and physiological importance. Mol. Interv. 3, 79–89 10.1124/mi.3.2.79 14993429

[B38] Obrig H. Neufang M. Wenzel R. Kohl M. Steinbrink J. Einhäupl K. (2000). Spontaneous low frequency oscillations of cerebral hemodynamics and metabolism in human adults. Neuroimage 12, 623–639 10.1006/nimg.2000.0657 11112395

[B39] Okamoto M. Dan H. Sakamoto K. Takeo K. Shimizu K. Kohno S. (2004). Three-dimensional probabilistic anatomical cranio-cerebral correlation via the international 10–20 system oriented for transcranial functional brain mapping. Neuroimage 21, 99–111 10.1016/j.neuroimage.2003.08.026 14741647

[B40a] Pagani M. Lombardi F. Guzzetti S. Rimoldi O. Furlan R. Pizzinelli P. (1986). Power spectral analysis of heart rate and arterial pressure variabilities as a marker of sympatho-vagal interaction in man and conscious dog. Circ. Res. 59, 178–193 10.1161/01.RES.59.2.178 2874900

[B40] Patel S. Katura T. Maki A. Tachtsidis I. (2011). Quantification of systemic interference in optical topography data during frontal lobe and motor cortex activation: an independent component analysis. Adv. Exp. Med. Biol. 701, 45–51 10.1007/978-1-4419-7756-4_7 21445768 PMC4038015

[B41] Rayshubskiy A. Wojtasiewicz T. J. Mikell C. B. Bouchard M. B. Timerman D. Youngerman B. E. (2013). Direct, intraoperative observation of ~0.1 Hz hemodynamic oscillations in awake human cortex: implications for fMRI. Neuroimage. [Epub ahead of print]. 10.1016/j.neuroimage.2013.10.044 24185013 PMC3961585

[B42] Rowley A. B. Payne S. J. Tachtsidis I. Ebden M. J. Whiteley J. P. Gavaghan D. J. (2007). Synchronization between arterial blood pressure and cerebral oxyhaemoglobin concentration investigated by wavelet cross-correlation. Physiol. Meas. 28, 161–173 10.1088/0967-3334/28/2/005 17237588

[B43] Saager R. B. Berger A. J. (2005). Direct characterization and removal of interfering absorption trends in two-layer turbid media. J. Opt. Soc. Am. A Opt. Image Sci. Vis. 22, 1874–1882 10.1364/JOSAA.22.001874 16211814

[B44] Saager R. B. Telleri N. L. Berger A. J. (2011). Two-detector Corrected Near Infrared Spectroscopy (C-NIRS) detects hemodynamic activation responses more robustly than single-detector NIRS. Neuroimage 55, 1679–1685 10.1016/j.neuroimage.2011.01.043 21256223

[B45] Sato H. Yahata N. Funane T. Takizawa R. Katura T. Atsumori H. (2013). A NIRS-fMRI investigation of prefrontal cortex activity during a working memory task. Neuroimage 83C, 158–173 10.1016/j.neuroimage.2013.06.043 23792984

[B46] Scholkmann F. Wolf M. Wolf U. (2013a). The effect of inner speech on arterial CO2 and cerebral hemodynamics and oxygenation: a functional NIRS study. Adv. Exp. Med. Biol. 789, 81–87 10.1007/978-1-4614-7411-1_12 23852480

[B47] Scholkmann F. Gerber U. Wolf M. Wolf U. (2013b). End-tidal CO2: an important parameter for a correct interpretation in functional brain studies using speech tasks. Neuroimage 66, 71–79 10.1016/j.neuroimage.2012.10.025 23099101

[B48] Scholkmann F. Kleiser S. Metz A. J. Zimmermann R. Mata Pavia J. Wolf U. (2013c). A review on continuous wave functional near-infrared spectroscopy and imaging instrumentation and methodology. Neuroimage. [Epub ahead of print]. 10.1016/j.neuroimage.2013.05.004 23684868

[B49] Söderström T. Stefanovska A. Veber M. Svensson H. (2003). Involvement of sympathetic nerve activity in skin blood flow oscillations in humans. Am. J. Physiol. Heart Circ. Physiol. 284, H1638–H1646 10.1152/ajpheart.00826.200 12679328

[B49a] Stauss H. M. Anderson E. A. Haynes W. G. Kregel K. C. (1998). Frequency response characteristics of sympathetically mediated vasomotor waves in humans. Am. J. Physiol. 274, H1277–1283 9575932 10.1152/ajpheart.1998.274.4.H1277

[B50] Steinbrink J. (2000). Near-Infrared-Spectroscopy on the Adult Human Head with Picosecond Resolution. Ph.D. thesis, FU Berlin, Berlin.

[B51] Tachtsidis I. Elwell C. E. Leung T. S. Lee C.-W. Smith M. Delpy D. T. (2004). Investigation of cerebral haemodynamics by near-infrared spectroscopy in young healthy volunteers reveals posture-dependent spontaneous oscillations. Physiol. Meas. 25, 437–445 10.1088/0967-3334/25/2/003 15132309

[B52] Tachtsidis I. Koh P. H. Stubbs C. Elwell C. E. (2010). Functional optical topography analysis using statistical parametric mapping (SPM) methodology with and without physiological confounds, in Oxygen Transport to Tissue XXXI, eds Takahashi E. Bruley D. F. (Boston, MA: Springer), 237–243 10.1007/978-1-4419-1241-1_34 PMC4038021 20204798

[B53] Tachtsidis I. Leung T. S. Chopra A. Koh P. H. Reid C. B. Elwell C. E. (2009). False positives in functional nearinfrared topography, in Oxygen Transport to Tissue XXX, Advances in Experimental Medicine and Biology, eds Liss P. Hansell P. Bruley D. F. Harrison D. K. (Springer), 307–314 10.1007/978-0-387-85998-9_46 19227487

[B54] Takahashi T. Takikawa Y. Kawagoe R. Shibuya S. Iwano T. Kitazawa S. (2011). Influence of skin blood flow on near-infrared spectroscopy signals measured on the forehead during a verbal fluency task. Neuroimage 57, 991–1002 10.1016/j.neuroimage.2011.05.012 21600294

[B55] Tanaka H. Katura T. Sato H. (2013). Task-related component analysis for functional neuroimaging and application to near-infrared spectroscopy data. Neuroimage 64, 308–327 10.1016/j.neuroimage.2012.08.044 22922468

[B56] Tong Y. Frederick B. D. (2010). Time lag dependent multimodal processing of concurrent fMRI and near-infrared spectroscopy (NIRS) data suggests a global circulatory origin for low-frequency oscillation signals in human brain. Neuroimage 53, 553–564 10.1016/j.neuroimage.2010.06.049 20600975 PMC3133965

[B57] Tong Y. Lindsey K. P. Frederick B. D. (2011). Partitioning of physiological noise signals in the brain with concurrent near-infrared spectroscopy and fMRI. J. Cereb. Blood Flow Metab. 31, 2352–2362 10.1038/jcbfm.2011.100 21811288 PMC3253380

[B58] Torrence C. Webster P. J. (1999). Interdecadal changes in the ENSO–monsoon system. J. Clim. 12, 2679–2690 10.1175/1520-0442(1999)012<2679:ICITEM>2.0.CO;2

[B59] Wabnitz H. Moeller M. Liebert A. Obrig H. Steinbrink J. Macdonald R. (2010). Time-resolved near-infrared spectroscopy and imaging of the adult human brain. Adv. Exp. Med. Biol. 662, 143–148 10.1007/978-1-4419-1241-1_20 20204784

[B60] Wabnitz H. Möller M. Liebert A. Walter A. Erdmann R. Raitza (2005). A time-domain NIR brain imager applied in functional stimulationexperiments, in Proc. SPIE, Vol. 5859, Photon Migration and Diffuse-Light Imaging II 58590H (Munich). 10.1117/12.632837

[B61] Zhang Q. Strangman G. E. Ganis G. (2009). Adaptive filtering to reduce global interference in non-invasive NIRS measures of brain activation: How well and when does it work? Neuroimage 45, 788–794 10.1016/j.neuroimage.2008.12.048 19166945 PMC2671198

[B62] Zhang X. Niu H. Song Y. Fan Y. (2012a). Activation detection in fNIRS by wavelet coherence, in Proc. SPIE, Vol. 8317, Medical Imaging 2012: Biomedical Applications in Molecular, Structural, and Functional Imaging, 831712 (San-Diego). 10.1117/12.911312

[B63] Zhang Y. Sun J. W. Rolfe P. (2012b). RLS adaptive filtering for physiological interference reduction in NIRS brain activity measurement: a Monte Carlo study. Physiol. Meas. 33, 925–942 10.1088/0967-3334/33/6/925 22551687

